# Genomic differentiation tracks earth-historic isolation in an Indo-Australasian archipelagic pitta (Pittidae; Aves) complex

**DOI:** 10.1186/s12862-019-1481-5

**Published:** 2019-07-24

**Authors:** Per G. P. Ericson, Yanhua Qu, Pamela C. Rasmussen, Mozes P. K. Blom, Frank E. Rheindt, Martin Irestedt

**Affiliations:** 1 Department of Bioinformatics and Genetics, Swedish Museum of Natural History, PO Box 50007, SE-104 05 Stockholm, Sweden; 2 Key Laboratory of Zoological Systematics and Evolution, Institute of Zoology, Chinese Academy of Sciences, Beijing, 100101 China; 3 Department of Integrative Biology and MSU Museum, Michigan State University, East Lansing, 48824 MI USA; 4 Bird Group, The Natural History Museum, Akeman Street, Tring, HP23 6AP UK; 5 Department of Biological Sciences, National University of Singapore, 14 Science Drive 4, Singapore, 119077 Singapore

**Keywords:** Allopatric divergence, de novo genome, Phylogenomics, *Pitta sordida*, Population genomics, Pleistocene glaciations, Sea level fluctuations

## Abstract

**Background:**

Allopatric speciation has played a particularly important role in archipelagic settings where populations evolve in isolation after colonizing different islands. The Indo-Australasian island realm is an unparalleled natural laboratory of biotic diversification. Here we explore how the level of earth-historic isolation has influenced genetic differentiation across the region by investigating phylogeographic patterns in the *Pitta sordida* species complex.

**Results:**

We generated a de novo genome and compared population genomics of 29 individuals of *Pitta sordida* from the entire distributional range and we reconstructed phylogenetic relationship using mitogenomes, a multi-nuclear gene dataset and single nucleotide polymorphisms (SNPs). We found deep divergence between an eastern and a western group of taxa across Indo-Australasia. Within both groups we have identified major lineages that are geographically separated into Philippines, Borneo, western Sundaland, and New Guinea, respectively. Although these lineages are genetically well-differentiated, suggesting a long-term isolation, there are signatures of extensive gene flow within each lineage throughout the Pleistocene, despite the wide geographic range occupied by some of them. We found little evidence of hybridization or introgression among the studied taxa, but *forsteni* from Sulawesi makes an exception. This individual, belonging to the eastern clade, is genetically admixed between the western and eastern clades. Geographically this makes sense as Sulawesi is not far from Borneo that houses a population of hooded pittas that belongs to the western clade.

**Conclusions:**

We found that geological vicariance events cannot explain the current genetic differentiation in the *Pitta sordida* species complex. Instead, the glacial-interglacial cycles may have played a major role therein. During glacials the sea level could be up to 120 m lower than today and land bridges formed within both the Sunda Shelf and the Sahul Shelf permitting dispersal of floral and faunal elements. The geographic distribution of hooded pittas shows the importance of overwater, “stepping-stone” dispersals not only to deep-sea islands, but also from one shelf to the other. The most parsimonious hypothesis is an Asian ancestral home of the *Pitta sordida* species complex and a colonization from west to east, probably via Wallacea.

**Electronic supplementary material:**

The online version of this article (10.1186/s12862-019-1481-5) contains supplementary material, which is available to authorized users.

## Background

Allopatric speciation, also referred to as vicariant speciation, appears to be the by far most common mode of speciation [1] and has played a particularly important role in archipelagic settings where populations evolve in isolation after colonizing different islands [2–4]. The Indonesian Archipelago, the Philippines, and New Guinea and its surrounding islands have been particularly affected by sea level fluctuations during the Pleistocene [5]. Cyclical reduction of polar ice sheets during interglacials has caused sea levels to rise and repeatedly broken up landmasses into islands. Formerly contiguous populations have thus been fragmented repeatedly, and if isolation lasted long enough, this has sometimes resulted in speciation. However, during ensuing glaciations sea levels have repeatedly fallen, sometimes by more than 120 m [5], facilitating contact and exchange of individuals. Naturally, these oscillations have affected only populations on islands that are part of shelf areas. Populations restricted to islands surrounded by deep water have been unaffected by the Pleistocene sea level fluctuations [6]. It can thus be postulated that a geographically widespread organism in this region will exhibit a phylogeographic pattern resulting from a mix of geological vicariance events, gene flow following secondary contact of previously isolated populations during periods of low sea levels, and incomplete lineage sorting in populations established by recent dispersal. Studying an organism distributed across the Indo-Australasian island realm, with its complex earth-historic and climatic history, can shed light on how these factors have influenced current genetic diversity.

**Fig. 1:**
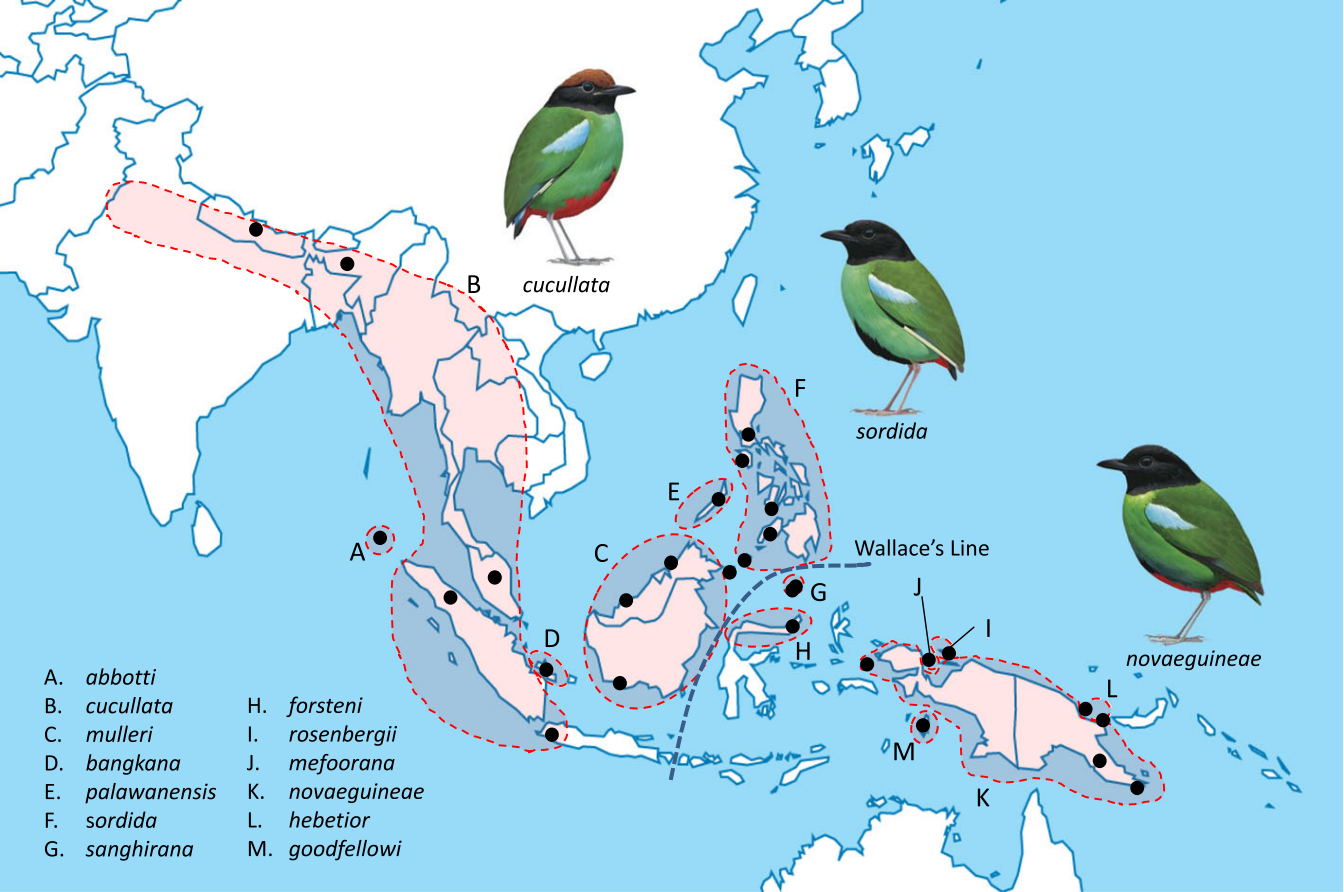
Geographic distribution of taxa included in the *Pitta sordida* species complex. Black dots indicate sampling localities (See Additional file 2: Table S12 for details). The map is drawn by us specifically for this paper and the bird images are reproduced with permission from Lynx Edicions

The hooded pitta (*Pitta sordida s.l.*) provides an excellent opportunity for such a study. Although long regarded a single species it is today recognized that this taxon consists of multiple populations with uncertain inter-relationships and unclear taxonomic status [7, 8]. Herein we call this group the *Pitta sordida* species complex. Members of this complex occur from northwestern India to New Guinea, spanning a distance of over 8,600 km (Fig. 1). Across its distribution this species complex exhibits considerable variation in morphology, vocalizations and body size, and numerous taxa have been described [9]. Plumage variation is primarily in forehead and crown color (from red-brown to black), the amounts of black, red and blue on belly and flanks, and the presence and size of a white wing patch [10]. Populations also vary in overall size, the average wing length being almost 20% larger in some populations than in others [11]. However, despite their large phenotypic variation no gradient in plumage or morphometry has been described within the wide geographic distribution of the *Pitta sordida* species complex. Also, from the rather sparse data available about ecological adaptations of these populations it is not possible to draw conclusions about geographic variation in, e.g., habitat choice, dietary preferences, or breeding behavior.

In this study we investigate evolutionary relationships and phylogeographic patterns within the *Pitta sordida* species complex to address how landmass isolation and glacial connections across the Indo-Australasian island realm may have contributed to the genetic diversity observed today. We hypothesize that different patterns of genetic differentiation can be predicted if the main driving force behind the diversification was the geological formation of the region, versus landscape-scale changes following Pleistocene sea-level fluctuations. Under the first scenario, we expect to find coalescence (divergence) time for the splits between taxa to be in broad agreement with tectonic movements and the formation of landmasses in the region, with limited subsequent gene flow. Under the second scenario, if Pleistocene land bridges were most important for the diversification of members of the *Pitta sordida* species complex we would assume gene flow would be more common and we would expect coalescence (divergence) times for the splits between several of the taxa to be congruent with the dating of recent glaciations. We interpret the observed patterns of spatio-temporal genetic differentiation in the light of the predictions following from these two scenarios.

## Results

### Phylogeny and population structure

**Fig. 2:**
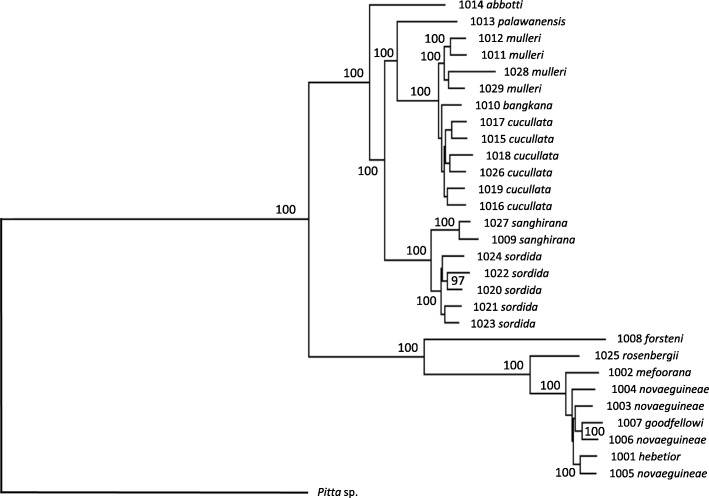
Phylogenetic relationships within the *Pitta sordida* species complex estimated from a concatenation of the mitochondrial genome (17,835 bp) and 23 nuclear genes (24,072 bp) using maximum-likelihood with RAxML. Numbers at nodes are bootstrap values (after 100 replicates). Only bootstrap values above 90 are shown

**Fig. 3:**
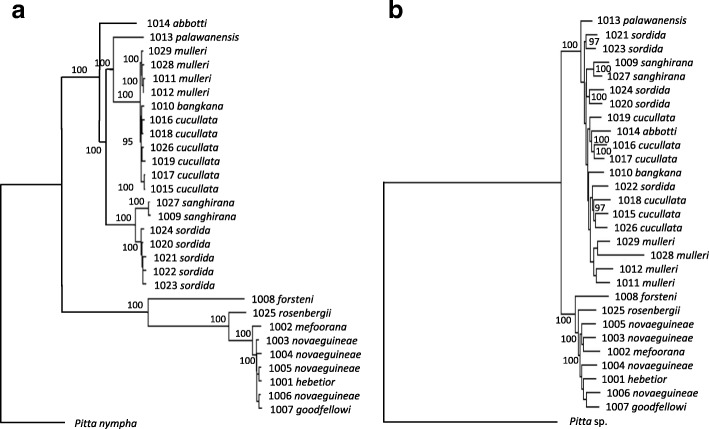
Phylogenetic relationships within the *Pitta sordida* species complex estimated from **a**) the mitochondrial genome (17,835 bp) and **b**) 23 nuclear genes (24,072 bp) using maximum-likelihood with RAxML. Numbers at nodes are bootstrap values (after 100 replicates). Only bootstrap values above 90 are shown

To infer phylogenetic relationships among the taxa we extracted several different data sets from the genomic sequences (see Additional file 2: Table S1 for details). The data sets possess varying properties and were subjected to a range of different analytical methods (maximum-likelihood analyses of nucleotides obtained from 23 nuclear genes as well as mitochondrial genomes, and neighbor-joining and principal component analyses of the ca. 2.1 million single nucleotide polymorphisms, SNPs). Regardless of data set and statistical method employed, these analyses yielded similar tree topologies within the *Pitta sordida* species complex (Figs. 2 and 3; Additional file 2: Table S2; Figures S1-S5). The most divergent phylogenetic signal came from the analyses of the 23 nuclear genes, both when analysed individually and after concatenation. However, the analyses of the nuclear genes in no case yielded high statistical support for tree topologies that are different from those obtained in the analyses of the mitochondrial genome and the SNP data. This suggests that the phylogenetic signal provided by each of the individual 23 nuclear genes is quite weak, possibly because these genes are evolving considerably more slowly than do those of the mitochondrion.

The analyses show a deep division of the *Pitta sordida* species complex corresponding to an eastern and a western group of taxa (Figs. 2 and 3; Additional file 2: Figures S1-S5). In the western group the individual from the population on the Nicobar Islands (*abbotti*) is sister to all other individuals. The latter individuals of the western group fall into two clades. The first clade comprises individuals from the Philippines (*sordida*) and Sangihe Islands (*sanghirana*), which are reciprocally monophyletic sister groups. The second clade of the western group is further divided into a Bornean sub-clade (*mulleri*) and one ranging over mainland Asia, Sumatra and Java (*cucullata* and *bangkana*). A few individuals end up in different phylogenetic positions depending on data type. One is the individual from Palawan (*palawanensis*) that groups with strong support with the *mulleri*-*cucullata*-*bangkana* clade in mitogenomic and concatenated mitogenomic plus nuclear ML analyses (Figs. 2 and 3). In most other analyses its position is unresolved when taking support values into account. The one exception is the neighbor-joining analysis of the SNP data in which *palawanensis* is positioned as sister to the *sordida*-*sanghirana* clade with strong support (Additional file 2: Figure S1). In the eastern group the individual from Sulawesi (*forsteni*) is recovered as sister to all other individuals with strong support across all analyses. There is little phylogeographic structure among the remaining individuals of the eastern group, except that all analyses strongly suggest that the individual from Biak Island (*rosenbergii*) is sister to all other individuals. Our data revealed no consistent phylogeographic patterns within the radiations in the Philippines (*sordida*), Borneo (*mulleri*), India, Nepal, Java, Sumatra and Malaysia (*cucullata*) or New Guinea (*novaeguineae*, *goodfellowi*, *hebetior* and *mefoorana*), respectively.

**Fig. 4:**
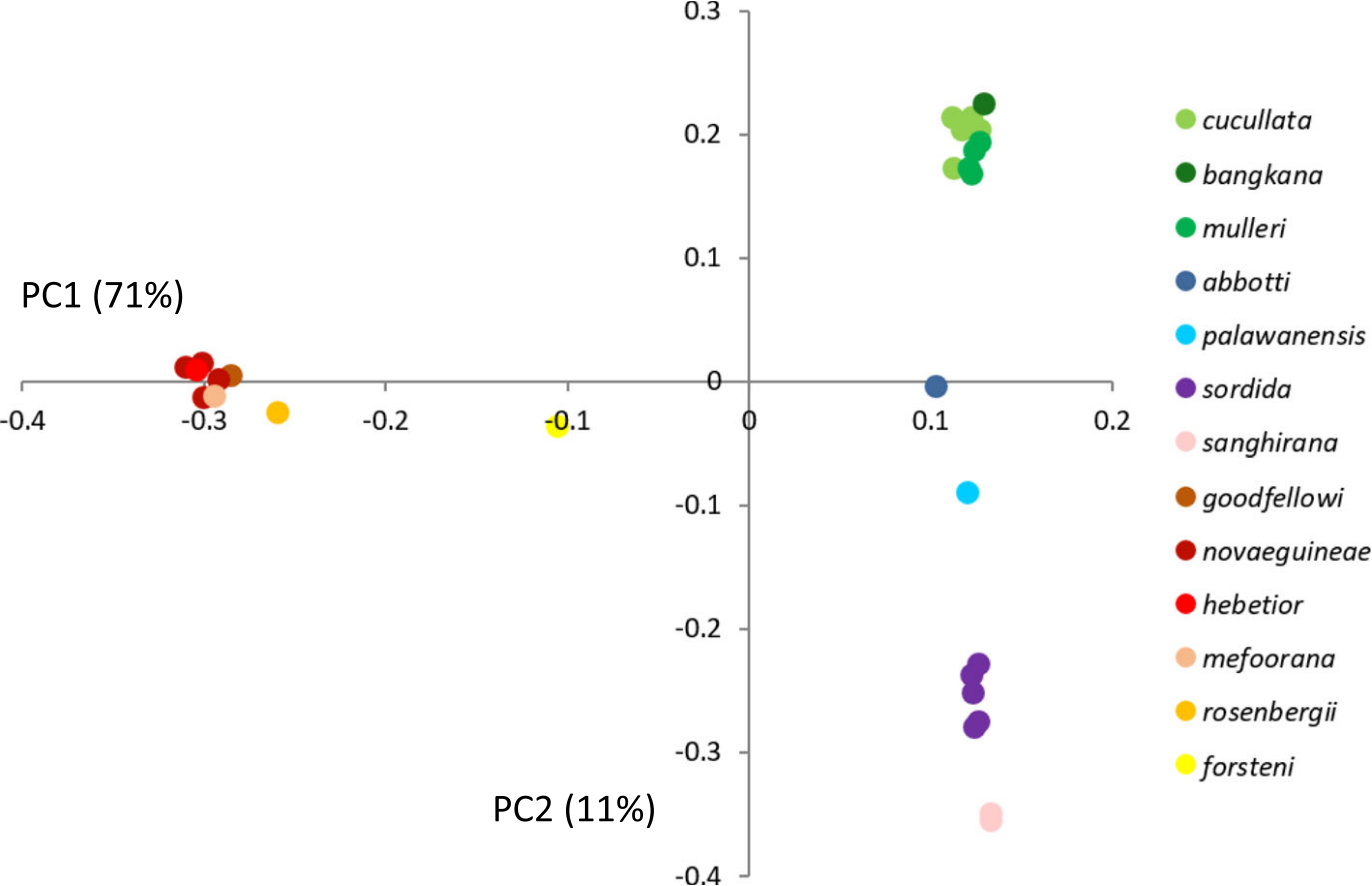
Principal component analysis (PCA) of the genomic variation among samples based on 2,193,399 SNPs. Only the first two components, together representing 82% of total variation, are shown. Note that *mefoorana*, *goodfellowi* and *hebetior* are included in *novaeguineae* in the main text

Plotting the two first principal components from principal component analysis (PCA) calculated from 2.1 million SNPs revealed a grouping that corresponds well with the phylogenetic results (Fig. 4). The first principal component (summarizing 71% of total variation) separates the eastern and the western groups. The individuals from the western group are distributed along the second principal component (amounting to 11% of total variation). A PCA including only *cucullata*, *mulleri* and *bangkana*, showed that *bangkana* falls in between *cucullata* and *mulleri* along the first principal component (summarizing 24% of total variation; Additional file 2: Figure S6).

**Fig. 5:**
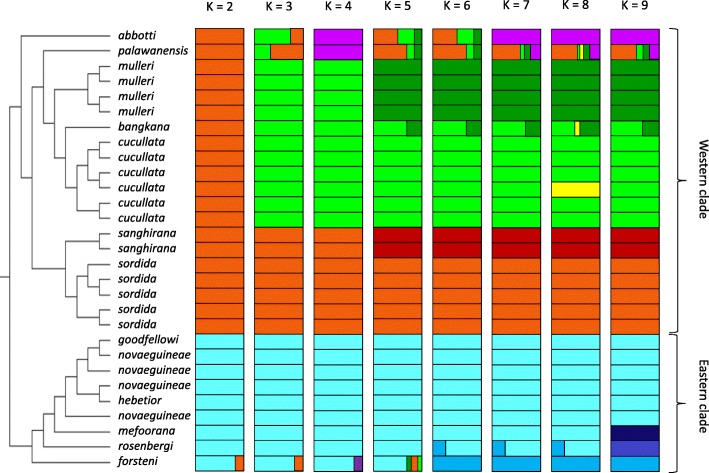
Genetic structure within the *Pitta sordida* species complex shown by ADMIXTURE plots for different numbers of ancestral clusters *K* = 2–9 based on 2,193,399 SNPs. Colored segments represent the individual’s estimated contribution to each subpopulation

ADMIXTURE analysis of population structure within the *Pitta sordida* species complex corroborates the distinct separation between the eastern and western clades regardless of *K* value (Fig. 5). As *K* = 2 resulted in the lowest cross-validation error among runs, it is thus inferred to be the best representation of the number of genetic groups in the data. The two major clades are thus genetically well differentiated, confirming that they have had a long, independent evolutionary history. With *K* = 2 only *forsteni* shows signs of admixture between the two clades, which may reflect shared, ancestral variation. For *K* values higher than two, *palawanensis* shows genomic admixture primarily between the *mulleri*-*cucullata*-*bangkana* and *sordida*-*sanghirana* clades (Fig. 5). The admixed nature of *palawanensis* most likely explains the alternative positions for this taxon in phylogenetic analyses depending on which data set is analysed. Similarly, for the individual from Bangka Island (*bangkana*), which in most phylogenetic analyses emerges in an unresolved position within the *mulleri*-*cucullata* clade (Figs. 2 and 3; Additional file 2: Figures S2-S3), ADMIXTURE analysis suggests that its genome is admixed between the *mulleri* and *cucullata* genomes (Fig. 5).

### Evolutionary history inference

We used G-PhoCS analysis to infer population divergence times, ancestral population sizes, and migration rates from a predefined phylogenetic tree. The results summarized in Additional file 2: Figure S7 and Additional file 2: Tables S3-S5 suggest that the initial split between the eastern and western clades dates back to almost 2 mya. According to this analysis, the earliest splits within each of these two clades took place in the mid-Pleistocene, when the Nicobar Islands (*abbotti*) and Sulawesi (*forsteni*) were colonized. According to G-PhoCS dating, the radiations in Malaysia, Sumatra and Borneo (*cucullata* and *mulleri*), Philippines (*sordida* and *sanghirana*) and New Guinea (*rosenbergii*, *novaeguineae*, *mefoorana*, *hebetior* and *goodfellowi*) all took place after the last ice age. As expected, isolated populations on smaller islands exhibit the smallest effective population sizes. Using G-PhoCS we also estimated post-divergence migration to be negligible both between the Philippine and Sundaic populations and between the New Guinean population and Biak Island as these were all low with the lower 95% HPD values virtually being zero (Additional file 2: Table S5).

G-PhoCS analysis resulted in surprisingly young divergence times between some clades within the *Pitta sordida* species complex, for example between the *sordida-sanghirana* and the *cucullata-mulleri* clades and within the New Guinean radiation. To determine how these results compare with ages calculated using a traditional mitochondrial clock approach we reconstructed an ultrametric tree based on the mitochondrial genome data applying a 1.05% substitution rate [12]. The molecular clock approach resulted in dates that were anywhere from roughly twice as old to one order of magnitude older than the G-PhoCS dates (Additional file 2: Table S6). To some degree this discrepancy may be due to incorrect assumptions about mutation rates or generation times unduly influencing inferences or oversimplified assumptions about the constancy of mutation rates across time. Given these discrepancies, we chose to interpret the estimated divergence times with caution.

### Introgression and gene flow

We used ABBA-BABA analyses to detect gene flow between non-sister taxa (Table 1; Additional file 2: Figures S8-S9). We found significant D-statistic indicating considerable gene flow in all tests that involved *palawanensis* on the one hand and *sordida* or *sanghirana* on the other. For all other combinations of taxa we only found low to very low D-statistic, although almost all tests returned very high Z-scores. However, herein we prefer to focus on the absolute values of the D-statistic instead of the Z-score as we found the latter to be highly correlated with the total number of ABBA and BABA sites regardless of whether their ratio was roughly constant (data not shown).

**Table 1:** Admixture signatures from D-statistic (ABBA-BABA test). Only comparisons resulting in a D-statistic higher than 0.05 or lower than − 0.05 are shown (see Additional file 2: Table S10; Figures S8-S9 for more information). Non-sister taxa with signatures of considerable gene flow are marked in bold

P1	P2	P3	O	nABBA	nBABA	D-statistic	Z-score
*cucullata*	***palawanensis***	***sordida***	*forsteni*	280′751	192′463	0.187	103.29
*cucullata*	***palawanensis***	***sanghirana***	*forsteni*	264′381	191′166	0.161	78.57
*mulleri*	***palawanensis***	***sanghirana***	*forsteni*	260′833	195′981	0.142	69.65
*sanghirana*	***sordida***	***palawanensis***	*forsteni*	210′343	180′080	0.078	45.00
***mefoorana***	*rosenbergii*	***forsteni***	*cucullata*	46′409	52′286	− 0.060	−12.41

We found high degrees of genomic erosion in the *Pitta sordida* species complex. In a majority of individuals we found long stretches of homozygosity (Additional file 2: Table S7; Figure S10). Most of the longest cumulative stretches of homozygosity were observed in populations that are assumed to have been long isolated on smaller islands, such as the Nicobars (*abbotti*), Numfor (*mefoorana*) and Biak (*rosenbergii*). The longest cumulative run of homozygosity (ROH) was observed in the individual from Sulawesi (*forsteni*). We also observed a weak, negative correlation (*r* = − 0.107) between the cumulative length of ROHs and island size (Additional file 2: Figure S11).

To distinguish introgression from incomplete lineage sorting in a four-taxon situation we applied *f*4 statistic on observed allele frequencies. We used the individual with the highest sequence coverage as representative of the studied population. For each individual we extracted between 9,009 and 16,952 biallelic SNPs from the multimarker data set (Additional file 2: Table S8). A high degree of these SNPs, between 9.4 and 17.9%, were variable within both pairs of sister taxa. The calculated *f*4 values did not differ significantly from zero in any of the studied four-taxon combinations, suggesting that the allele frequencies observed may be explained by incomplete lineage sorting alone, without introgression.

### Demography fluctuation estimations

Testing demographic expansion of different lineages using DnaSP showed that the null hypothesis of an expanding population could not be rejected for ancestral populations (Additional file 2: Table S9). This differs from the populations that occupy the larger landmasses today. In several of these (*cucullata* in Malaysia, Sumatra and Java, *sordida* in the Philippines, and *novaeguineae*, *mefoorana*, *hebetior* and *goodfellowi* in New Guinea) the analyses indicated that populations are in equilibrium (Additional file 2: Table S9). Only for *mulleri* in Borneo a reduction in population size is the best explanation of the observed data. The PopSizeABC analysis also shows a decline of the *mulleri* population size over the last ca. 10,000 years (Additional file 2: Figure S12).

## Discussion

### The relative roles of geological vicariance events and Pleistocene Sea level fluctuations in shaping the genetic differentiation

The impetus of the study was to investigate to what degree landmass isolation and glacial connections across the Indo-Australasian island realm explain the genetic diversity observed across the *Pitta sordida* species complex. We hypothesize that if the main driving force behind the diversification was the geological formation of the region we would expect to find coalescence (divergence) times for splits between taxa to be in broad agreement with tectonic movements and the formation of landmasses in the region. Under the alternative scenario, that landscape changes following fluctuations in sea level during the Pleistocene explain most patterns of genetic differentiation among hooded pittas, we would expect coalescence (divergence) times for the splits between several of the taxa to be congruent with the timing of recent glaciations. However, a considerable discrepancy observed between the different dating methods employed makes the evaluation less straightforward. The classical genetic dating method using molecular clock estimates for cytochrome *b* yields older dates, by orders of magnitude, than a genome-wide, coalescent-based method (G-PhoCS). Regardless of the dating method employed we infer the earliest split within this complex into an eastern and a western group to have occurred by the early Pleistocene. This dating is inconsistent with the idea that tectonic movements of landmasses in the region have been important in the diversification of the *Pitta sordida* species complex, as these took place well before the Pleistocene [13].

**Fig. 6:**
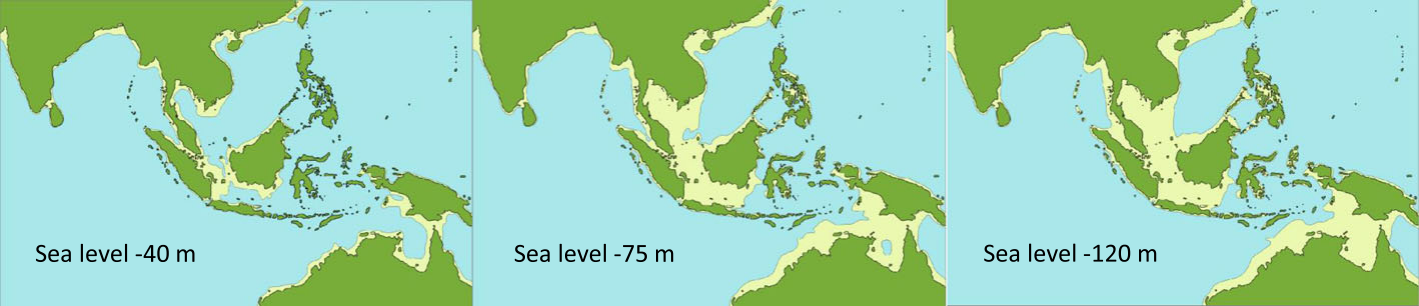
Effects of Late Pleistocene sea level fluctuations in the Indo-Australasian region. Vast quantities of sea water was locked up in continental ice sheets during the glacial periods. As a consequence, sea levels intermittently sank up to 120 m exposing large landmasses (yellow) within the Sunda and Sahul Shelves, respectively. This allowed dispersal of faunal and floral elements between shelf areas that were isolated during the interglacials. The figure is based on maps published in [5] with permission from the author

We thus conclude that geological vicariance events cannot explain the current genetic differentiation in the *Pitta sordida* species complex. The timing of the radiation in the Pleistocene suggests that the glacial-interglacial cycles may have played a major role therein, especially the sea level fluctuations of up to 120 m between glacial and interglacial periods [5], which have resulted in intermittent land bridges (Fig. 6). Whenever the sea level reaches its low point (such as during the Last Glacial Maximum ca 18 kya), the geography of the region is dominated by two large sub-continental landmasses, the Sunda Shelf (to which Borneo, Java, Sumatra and peninsular Malaysia belong) and the Sahul Shelf (which includes New Guinea, Australia and nearby islands). Between these two Quaternary landmasses, there is a large number of deep-sea islands that today form Wallacea and the Philippine archipelago.

The early Pleistocene dating of the split between the eastern and western clades of hooded pittas suggests overwater colonization of one of the shelves, most likely via Wallacea. The two species that are phylogenetically closest to the *Pitta sordida* species complex, *Pitta nympha* and *Pitta moluccensis* [14], both breed in mainland Southeast Asia. The most parsimonious hypothesis is thus an Asian ancestral home of the complex and a colonization from west to east.

The phylogeographic analysis suggests that over-water dispersal must have been common when members of the *Pitta sordida* species complex dispersed in the region. In most cases we can assume short-distance, “island-hopping” dispersal events in periods of low sea levels, but from the data we can also infer multiple dispersal events over substantial stretches of water. The most significant is the colonization of the southern Nicobar Islands (*abbotti*). The Nicobars have always been unconnected to any larger landmass, and today the closest hooded pitta population is on northern Sumatra, ca. 170 km away. The colonization of the Nicobars may have occurred in the mid Pleistocene (Additional file 2: Table S4; Figure S7) and today the *abbotti* population is well differentiated genetically, morphologically, and vocally. The fact that this population exhibits among the largest cumulative runs of homozygosity observed among all samples studied herein (Additional file 2: Figure S10) suggests that it has been isolated from the founding population for a long time, perhaps even since the time of the initial colonization. The indication of inbreeding is strengthened by the observation that its effective population size has been low for as long as can be inferred. G-PhoCS and PopSizeABC analyses suggest that the effective population of *abbotti* has remained low, at most around 200–400 individuals, for the last ca. 100 ky (Additional file 2: Table S3; Figure S12).

A “stepping-stone” colonization of Wallacea must have been highly significant in the further diversification of the hooded pitta. Borneo and Sulawesi have always been separated by a deep-water strait (the Makassar Strait) that constitutes part of the most important biogeographical divide in this region, Wallace’s Line (see Fig. 1). The line is named after Alfred Russel Wallace who during his travels here in the mid-nineteenth century observed prominent differences in the fauna and flora in the eastern and western parts of the Indo-Australasian archipelago, respectively. It is striking that the deepest split within the *Pitta sordida* species complex coincides with the dispersal across this divide. However, the faunal and floral elements for which Wallace’s Line constitutes a major boundary are typically much older than the radiation of hooded pittas. Furthermore, published data does not provide evidence that populations within the *Pitta sordida* species complex to the west and east of the line differ in their habitat choice, dietary preferences, breeding behavior, etc. We thus do not believe that the climatological and other differences between the western and eastern parts of the Indo-Australasian archipelago have been significant for shaping genetic structure of this species complex. Dispersal across the Makassar Strait, assumed to have occurred in the mid-Pleistocene, doubtless established the hooded pittas on both sides of the Wallace’s Line and facilitated their further dispersal and differentiation.

There is a puzzling gap in the present-day distribution of hooded pittas around the Moluccas (incl. Halmahera, Seram, Buru, etc.), which are situated halfway between Sulawesi and New Guinea (Fig. 1). The large geographic distance (ca. 700 km mostly over water) between Sulawesi and the closest New Guinean populations renders direct contact improbable. The former existence of a now-extinct Halmahera population could thus explain the observed gene flow between Sulawesi and New Guinea (Additional file 2: Table S10). A significant D-statistic does not necessarily indicate direct introgression between genomes under study, but could also signify indirect gene flow via an extinct (unsampled) population [15–17]. Several other islands that have long been isolated from larger landmasses hosting populations of the *Pitta sordida* species complex, yet all those studied herein seem to be part of rapid and probably young radiations (see below).

### Recent gene flow obscures older phylogeographic patterns

The onset of glacial cycles has resulted in dramatic landscape changes across the Indo-Australasian island realm (Fig. 6). During glacial times when the sea level has been up to 120 m lower than today, many faunal and floral elements have readily dispersed between islands via land bridges. Various populations of the *Pitta sordida* species complex that most likely were isolated during the interglacials came into contact during past glacials when the sea level dropped. As a result, there has been intermittent gene flow across both the Sunda Shelf and across the Sahul Shelf throughout the Pleistocene. Consistent with this, we find few examples of older phylogeographic structure within the *cucullata*-*mulleri*-*bangkana*-group, and within the *novaeguineae*-*mefoorana*-*hebetior*-*goodfellowi*-group (Fig. 2; Additional file 2: Figures S1-S5). This is somewhat surprising given that each of these groups have wide geographic distributions of which we have sampled all parts (Fig. 1). However, the correlation between genetic and geographic distances among samples is not high in either the western or eastern groups (Additional file 2: Figures S13-S14). It seems clear that cyclical fluctuations in gene flow following from contractions and expansions of these populations have efficiently blurred most ancient genetic structure that may have existed.

**Fig. 7:**
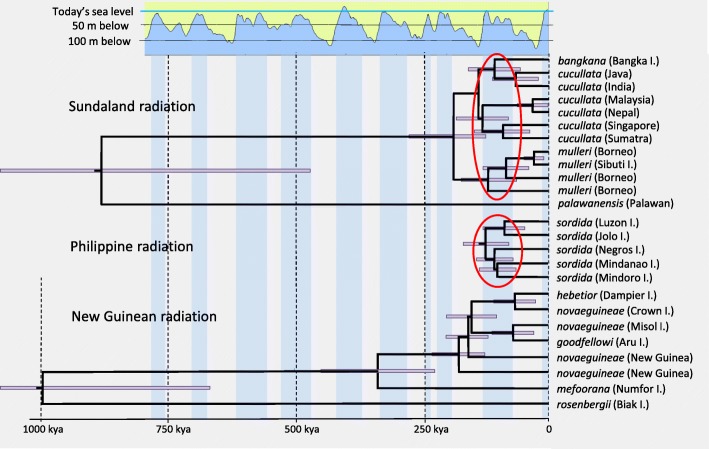
Relationship between sea level fluctuations during the Late Pleistocene and the diversifications within three radiations of hooded pittas. The trees are extracted from the mitochondrial genome phylogeny (Additional file 2: Figure S3a) and dated by an assumed 2.1% per million year divergence between taxa. Purple bars indicate 95% credibility intervals. Interglacial periods with sea levels less than 50 m below present time are marked in blue (data from [18]). The populations in Sundaland (including Borneo, Java, Sumatra and peninsular Malaysia) and the Philippines seemingly underwent radiations during the last interglacial following the fragmentation of these regions caused by the raising sea level

The lack of phylogeographic structure within the radiations in the Sunda and Sahul Shelves may indicate that these are not very old. Applying a mitochondrial clock model [12] to these radiations suggests that the two Sunda Shelf radiations (the clade including *cucullata-mulleri-bangkana* and the clade with *sordida*) both diversified when subpopulations were isolated by the rising sea levels during the last interglacial (Fig. 7). A similar correlation between rising sea level and genetic diversification cannot be observed within the New Guinean radiation. An obvious exception to the lack of genetic structure across New Guinea is the *rosenbergii* population on the small (2,455 sq. km) island of Biak that in all analyses turned out to be sister to all other members of the New Guinean clade (Figs. 2 and 3; Additional file 2: Figures S1-S5) despite Biak’s proximity to the populations on Numfor (only ca. 20 km) and mainland New Guinea (ca. 40 km). Also the population on nearby Numfor Island (*mefoorana*) falls outside the other New Guinean members of the *Pitta sordida* species complex (minus *rosenbergii*) in several analyses. Gene flow through migration between the islands of Biak and Numfor, as well as between these and mainland New Guinea, is low (Additional file 2: Table S10). This observation underscores the importance of sea level fluctuations in generating opportunities for gene flow considering that both Biak and Numfor are surrounded by deep sea with no Quaternary land bridges to New Guinea.

The case of New Guinea is intriguing. It is unknown for how long members of the *Pitta sordida* species complex have lived in New Guinea, but we found no trace of ancient phylogeographic structure, despite having sampled all New Guinean populations that have historically been suggested to be morphologically distinct (*novaeguineae*, *hebetior* and *goodfellowi*). The almost non-existent phylogeographic structure may suggest considerable gene flow across this huge island, including with the smaller shelf islands surrounding it. Alternatively, the current lack of genetic structure could reflect a rapid and recent dispersal into novel areas.

### Evidence of hybridization and introgression

There is little evidence of hybridization or introgression among the studied taxa (Table 1; Fig. 5). A clear case is the individual of *forsteni*, which is admixed between the western and eastern clades. This also makes geographic sense as the *forsteni* population, which belongs to the eastern clade of the *Pitta sordida* species complex, lives on Sulawesi and thus not far from Borneo that houses the *mulleri* population belonging to the western clade. Two more samples in the western clade, *bangkana* from Bangka Island and that of *palawanensis* from Palawan, exhibit an admixed genomic signature at *K* values larger than two. The *bangkana* individual appears to be admixed between nearby populations of Sumatra and Borneo (*mulleri*) and the migratory population in mainland Asia (*cucullata*) (Fig. 5). The *bangkana* population may have formed when wintering *cucullata* and sedentary *mulleri* individuals were isolated in the Bangka and Belitung islands following rising sea levels. This is likely to have happened during one of the latest interglacials, as the population has remained genetically distinct to the present day. The taxon *palawanensis* is being admixed between *sordida* (which occupies the Philippines just north of Palawan) and *mulleri* (which occupies Borneo to the southwest). Although this makes full geographic sense, the genetic uniqueness of the Palawan population indicates a low level of gene flow despite the proximity to the populations in both the Philippines and Borneo.

### Long-distance migration is rare in the *Pitta sordida* species complex

Most populations within the *Pitta sordida* species complex are either sedentary or undertake short seasonal movements. Only the taxon *cucullata* is a long-distance migrant, wintering in Sumatra and peninsular Malaysia but breeding as far away as the Himalayas of India, Nepal and China. In the winter *cucullata* occurs alongside members of its sedentary sister taxon *mulleri*. The two species most closely related to the *Pitta sordida* species complex, *Pitta nympha* and *P. moluccensis* [10, 14], are long-distance migrants. If one assumes that the ancestor of the *Pitta sordida* species complex was migratory, this behavior was independently lost in all lineages except *cucullata*. An alternative scenario would entail migratory behavior to be lost in the ancestor but re-evolved in *cucullata*. It is difficult to differentiate between these two scenarios as migratory behavior may be a labile trait, easy to lose and to re-gain. Also, many species are known to be partially migratory, i.e. migratory only in sub-optimal regions but resident in others. This partial migratory state is perhaps an apt description for the *cucullata* - *mulleri* clade, which is genomically compact but consists of both migratory and non-migratory populations. Under this scenario, migratory behavior may have been inherited from an ancestor and retained by some but not all populations of hooded pittas throughout their history.

## Conclusions

We found that geological vicariance events cannot explain the current genetic differentiation in the *Pitta sordida* species complex. Instead, the glacial-interglacial cycles may have played a major role therein. During glacials the sea level could be up to 120 m lower than today and land bridges formed within both the Sunda Shelf (to which Borneo, Java, Sumatra and peninsular Malaysia belong) and the Sahul Shelf (which includes New Guinea, Australia and nearby islands). These land bridges intermittently permitted dispersal of floral and faunal elements, which may have contributed to obscure older phylogeographic patterns within each shelf. The geographic distribution of hooded pittas shows the importance of overwater, “stepping-stone” dispersals not only to deep-sea islands, but also from one shelf to the other. The most parsimonious hypothesis is an Asian ancestral home of the *Pitta sordida* species complex and a colonization from west to east, probably via Wallacea. There is little evidence of hybridization or introgression among the studied taxa, but one exception is *forsteni* from Sulawesi. This individual, belonging to the eastern clade, is genetically admixed between the western and eastern clades. This makes geographic sense as Sulawesi is not far from Borneo that houses a population of hooded pittas that belongs to the western clade.

## Methods

A full description of methods is given in Additional file 1.

### Sampling

We sampled all traditionally recognized taxa in the *Pitta sordida* species complex [9, 11, 14, 19–22] (Fig. 1; Additional file 2: Tables S11-S12) across its entire distribution, from Nepal to eastern New Guinea. For the sake of convenience, we herein follow the taxonomy of Erritzoe & Erritzoe [11] who recognized one species, *Pitta sordida*, with 13 subspecies. Although all these subspecies were sampled, we include *goodfellowi* and *hebetior* within *novaeguineae* in the discussion of the populations in New Guinea if not explicitly stated otherwise. The rationale for this is that these three subspecies were found to be genetically inseparable in our analyses.

### Sequencing, reference mapping and variant calling

We de novo sequenced a sample of an individual of *Pitta sordida cucullata* found freshly dead in Bukit Batok Nature Park in Singapore after collision with a window. It was assigned to the migratory subspecies *cucullata* based on its brown crown. The resident Sundaic subspecies *mulleri*, which has a black crown, is not yet known from Singapore. Tissue aliquots of the specimens were deposited at the Lee Kong Chian Natural History Museum (Singapore). For another 28 individuals we extracted DNA from toe-pads sampled from museum study skins (Additional file 2: Table S12). An Illumina HiSeqX platform at the National Genomics Institute in Stockholm was used both for de novo sequencing and whole-genome resequencing. Reads were processed using a custom designed, clean-up workflow that is available at https://github.com/mozesblom. We mapped the reads against several references to construct data sets of different characteristics for phylogenetic analyses, e.g., fast evolving mitochondrial genes and more slowly evolving nuclear genes (Additional file 2: Table S1). As expected, mapping coverage varied considerably among data sets and individuals (Additional file 2: Table S13). The mitochondrial genome had a mean mapping coverage of 998x while mapping to the de novo genome obtained in this study gave a mean coverage of 5.7x. SNPs were called from the genome BAM-files using two different workflows (Samtools and GATK), but for analyses we used only those variants that were identified by both workflows (see Additional file 1 for a more detailed description of the initial bioinformatics).

### Phylogenetic analyses and population genetic structure

We estimated best-fit maximum-likelihood trees with RAxML [23] both individually for each of the 23 nuclear genes and the mitochondrial genome, for the concatenated data sets consisting of all 23 nuclear genes for which taxon-complete alignments were available, and for this latter data set combined with the mitochondrial data set (totaling 41,907 bp before filtering) (see Additional file 2: Table S1 for more details). We estimated phylogenetic relationships from SNP data using a neighbor-joining algorithm in TreeBest [24]. We also used SNPs to infer population genetic structure both by principal component analyses using smartpca in EIGENSOFT v. 6.1.4 [25] and with the clustering algorithms FRAPPE v. 1.1 [26] and ADMIXTURE v. 1.3 [27]. The degree to which genetic differentiation can be explained by geographic distance between sampling localities was assessed by plotting the genomic distance (*p*-distances) between each pair of individuals against their geographic distance.

### Demographic history and genetic admixture

Demographic history of the *Pitta sordida* species complex, including population divergence times, ancestral population size and migration rates, was inferred using the Generalized Phylogenetic Coalescent Sampler (G-PhoCS) [28] and a data set consisting of 1,569 segments of 202 bp each. The data were derived from McCormack et al. [29] who obtained a total of 316 kb of nucleotides from the flanking regions of 1,572 different UCE (ultra-conserved elements) loci distributed across the genome of the banded pitta *Pitta guajana*. The flanking regions around the UCEs are characterized by having a high variability and evolving neutrally [30]. We downloaded the concatenated data set from the Dryad data package [31] and used it as a mapping reference. After alignment we arbitrarily divided the data set into 1,569 segments consisting of 202 bp each. Burn-in and convergence of each run in G-PhoCS were determined with TRACER v. 1.7 [32]. We repeated the analysis with six separate runs to obtain reliable and stable estimates for the demographic parameters. Posterior distributions of τ (coalescent branch lengths) and θ (ancestral scaled population sizes) were re-calculated to divergence times in units of years, effective population sizes, and migration rates by scaling with a neutral mutation rate of 4.6*10^− 9^ [33] and a generation time of 4.2 years [34]. The amount of gene flow was estimated from the mean number of migrants per generation (M_s-t_) for the migration bands inferred by G-PhoCS using the predefined tree topology in Fig. 2 (after pruning *palawanensis* from the tree, see text in Additional file 1 for details).

We tested for differential gene flow between populations by applying multi-population ABBA-BABA tests in ANGSD [35] and used the *f*4-statistic [36] to distinguish introgression from incomplete lineage sorting based on allele frequencies of four populations. Additionally, intra-individual runs of homozygosity (ROH), i.e. uninterrupted stretches of homozygous genotypes resulting from parents transmitting identical haplotypes to their offspring, were estimated in PLINK v. 1.9 [37] to assess the levels of inbreeding. We used PopSizeABC, an approximate Bayesian computation pipeline [38], to estimate temporal variation in effective population size (N_e_) for individuals in each lineage. These statistics are first calculated for an empirical SNP data set and then compared with the corresponding statistics calculated from a large number of simulated data sets. The summary statistics for the empirical data sets are then compared with 400,000 simulated data sets to identify the simulations that are most similar. In another analysis we used DnaSP v. 6.11.01 [39] to test the null hypothesis of a recent expansion of a given population by comparing observed and expected distributions of differences between pairs of haplotypes and applying neutrality tests, the raggedness index r_g_ [40] and R_2_ (which is particularly suitable for small sample sizes [41]).

## Additional files

Additional file 1: Detailed description of material and methods. (PDF 459 kb)

Additional file 2: Supplementary tables and figures. **Table S1** Data sets used for reference mapping. **Table S2** Comparison of different tree topologies reconstructed in the phylogenetic analyses of different data sets and by employing different methods. Note that several topologies are incompatible with each other. **Table S3** Calculated effective population sizes (Ne) inferred by G-PhoCS using the predefined tree topology in Fig. 2 (after pruning palawanensis from the tree, see text in Additional file 1 for details) and after calibrating the estimated parameter θ assuming an average mutation rate of 4.6*10^− 9^substitutions per generation and an average generation length of 4.2 year. **Table S4** Calculated divergence times (T) inferred by G-PhoCS using the predefined tree topology in Fig. 2 (after pruning *palawanensis* from the tree, see text in Additional file 1 for details) and after calibrating the estimated parameter τ assuming an average mutation rate of 4.6*10^− 9^ substitutions per generation and an average generation length of 4.2 year. **Table S5** Estimates of mean number of migrants per generation (Ms-t) for the migration bands inferred by bands inferred by G-PhoCS using the predefined tree topology in Fig. 2 (after pruning *palawanensis* from the tree, see text in Additional file 1 for details). **Table S6** Comparisons of estimates of divergence times for major clades within the “*Pitta sordida* species-complex” using a molecular clock approach in Beast2 and genomic mutation rates in G-PhoCS. In the G-PhoCS analysis we used two different estimates of the mutation rate in passerines; 3.0*10^− 9^(Zhang et al. 2014) and 4.6*10^− 9^(Smedset al. 2016). **Table S7** Individuals and scaffolds in which longer (> 250 Mb) stretches of homozygosity was observed. **Table S8** The *f*4-statistic was calculated in four-taxon comparisons to test different hypotheses of introgression. **Table S9** Analysis of mismatch distribution in populations within the “*Pitta sordida* species-complex” from which two or more individuals were sampled. **Table S10** Admixture signatures from D-statistic (ABBA-BABA test). **Table S11** Alternative taxonomic classifications of the taxa included in the “*Pitta sordida* species-complex”. **Table S12** Specimen data for the samples used in the study. **Table S13** Mapping coverage estimated with genomecovin BEDToolsv.2.26.0. **Figure S1** Phylogenetic relationships within the “*Pitta sordida*” species-complex reconstructed with a neighbor-joining analysis of 2,193,399 high-quality SNPs. **Figure S2** Phylogenetic relationships within the “*Pitta sordida*” species-complex inferred with MP-EST. **Figure S3** Phylogenetic relationships within the “*Pitta sordida*” species-complex inferred with MP-EST. **Figure S4** Posterior distribution of trees obtained in a Bayesian analysis using SNAPP based on 22,074 biallelicSNPs shared across the taxa in the western clade. **Figure S5** Posterior distribution of trees obtained in a Bayesian analysis using SNAPP based on 17,810 biallelicSNPs shared across the taxa in the eastern clade. **Figure S6** Principal component analysis (PCA) of the genomic variation among the samples assigned to the taxa *mulleri*, *cucullata*and *bangkana*. **Figure S7** Divergence times and effective population sizes (Ne) inferred by G-PhoCSusing the predefined tree topology from Fig. 2 (after pruning *palawanensis*, see Additional file 1). **Figure S8** D-statistic (ABBA-BABA) for the tested comparisons within the western clade. **Figure S9** D-statistic (ABBA-BABA) for the tested comparisons within the eastern clade. **Figure S10** Cumulative size of the genome fraction allocated in runs of homozygosity (> 250 kb) per sample. **Figure S11** Cumulative size of the genome fraction allocated in runs of homozygosity (> 250 kb) plotted against the size of the island from where the sample was collected. **Figure S12** Variation in effective size (Ne) in populations represented by more than one individual inferred with the approximate Bayesian computation pipeline PopSizeABC using 1,304,779 SNPs. **Figure S13** Scatterplot of the genetic (p-distances) and geographic distances (km) between all pairs of samples in the western clade. **Figure S14** Scatterplot of the genetic (p-distances) and geographic distances (km) between all pairs of samples in the eastern clade. (PDF 1643 kb)

## Data Availability

The datasets supporting the conclusions of this article are available in the Dryad repository, doi: 10.5061/dryad.j78f467. Raw Illumina sequences are deposited in Sequence Reads Archive, National Center for Biotechnology Information, SRA accession PRJNA552359 (https://www.ncbi.nlm.nih.gov/sra/PRJNA552359).

## Abbreviations

bp: Base pair
ky: Thousand years
SNP: Single nucleotide polymorphisms
UCE: Ultra-conserved element
