# Supplementary material for: Genomic differentiation tracks earth-historic isolation in an Indo-Australasian archipelagic pitta (Pittidae; Aves) complex
Source: BMC Evol Biol. 2019 Jul 24;19:151. doi: 10.1186/s12862-019-1481-5 (PMC6657069; doi:10.1186/s12862-019-1481-5)
Supplement: Supplementary file 1 — Detailed description of material and methods. (PDF 459 kb) [file 12862_2019_1481_MOESM1_ESM.pdf]

BMC Evolutionary Biology

Genomic differentiation tracks earth-historic isolation in an Indo-Australasian archipelagic pitta (Pittidae; Aves) complex

Per G.P. Ericson, Yanhua Qu, Pamela C. Rasmussen, Mozes P.K. Blom, Frank E. Rheindt & Martin Irestedt

Additional file 1: Detailed description of material and methods.

1. *Sampling*

We have sampled all traditionally recognized taxa in the "*Pitta sordida* species-complex" (Mayr 1979; Lambert & Woodcock 1996; Erritzoe & Erritzoe 1998; Erritzoe 2003; del Hoyo et al. 2018a, 2018b; Erritzoe & Kirwan 2018) (Table 1, Fig. 1, Suppl. Tables S11-S12). In this paper we will use the term "*Pitta sordida* species-complex" for all these taxa. We have sampled across the whole distribution of the "*Pitta sordida* species-complex", from Nepal to eastern New Guinea. For clarity we herein follow the taxonomy of Erritzoe & Erritzoe (1998) who recognized one species, *Pitta sordida*, with thirteen subspecies. Although all these subspecies were sampled, we include *goodfellowi* and *hebetior* in *novaeguineae* in the discussions of the populations in New Guinea if not explicitly stated otherwise. The rationale for this is that these three subspecies were found to be genetically inseparable in our analyses.

2. *Sequencing of a de-novo genome of* Pitta sordida

DNA extracted from a fresh sample of *Pitta sordida cucullata* collected in Bukit Batok Nature Park in Singapore (Suppl. Table S12) was used for de-novo sequencing. Four DNA libraries, one short-insert-sized, paired-end (180 bp) and three mate-pair (3 and 5-8 kb) DNA libraries, were sequenced on an Illumina HiSeq X platform at the National Genomics Institute. Low quality and duplicated reads were filtered out before assembly. Unfortunately the 5-8 kb mate-pair library did not work well despite having tried to sequence it twice. The final genome was assembled by Science for Life Laboratory (National Genomics Institute, Stockholm) using their best-practice analysis for *de novo* assembly and assembly evaluation. Three different assemblers were used and their performances were evaluated by aligning a subset of Illumina reads back to the assembled sequence. The lowest number of scaffolds (12,146) and the highest scaffold N50 values (405 kb) were obtained by the ALLPATHS_LG assembler and this assembly was used for the downstream analyses.

3. *Whole genome re-sequencing from museum study skins*

Genomic DNA was extracted from toe-pad samples of 28 museum study skins kept at the American Museum of Natural History and Naturalis, Leiden (Suppl. Table S12). The genome library preparation for Illumina high-throughput sequencing followed the protocol published

by Meyer and Kircher (2010). The libraries were pooled and sequenced on an Illumina HiSeqX platform at the National Genomics Institute. Applying a whole genome shotgun strategy generated a total of 4.4 Gb of paired-end reads of 151 bp length.

To avoid reads with artificial bias in the process of library construction and sequencing (i.e. low quality reads, which mainly resulted from base-calling duplicates and adapter contamination), we used a custom designed, clean-up workflow that is available at https://github.com/mozesblom. The workflow uses Super Deduper (Petersen et al. 2015) to remove read duplicates introduced by the PCR amplification. TRIMMOMATIC v. 0.32 (Bolger et al. 2014) was used to remove sequencing adapters and to filter away low-quality reads. The reads to be retained should exceed 30 bp in length. Paired-end reads were merged using PEAR (Zhang et al. 2014) if they overlap with at least 20 bp (with the probability threshold set to 0.001). As the samples sequenced in this study are from old museum study skins the DNA is quite degraded. As a result most reads were short and only a low proportion of them (on average 1-2%) could be paired. In the reference mapping we used both the paired and the unpaired reads. The workflow also removed low-complexity reads (single or merged), i.e. reads where more than half of the base pairs consisted of the same nucleotide (A, T, C, G or N). The overall quality and length distribution of sequence reads were inspected prior and post the clean-up workflow using FASTQC v. 0.11.5 (Andrews 2010).

In the phylogenetic analyses we also included the individual for which a de-novo genome had been sequenced. In the analyses we used ca. 20% of the paired-end reads sequenced for this individual in order to use a similar amount of reads as what were obtained from the museum skin samples.

4. *Reference mapping*

It can be postulated that different parts of the genome will show conflicting signatures due to their inherited characteristics. For example, incomplete lineage sorting and recent admixture among previously isolated lineages may leave different fingerprints in fast evolved gene (e.g. mtDNA) and slow evolved genes (e.g. nuclear genes). Herein we use four datasets with different parts of the genomic architecture to study the genetic history of the hooded pitta. First, the entire nuclear genome was mapped using the ALLPATH_LG assembly of the *Pitta sordida* genome (see above). Second, mitochondrial data (evolving relatively fast) was obtained by mapping against the complete mitochondrial genome of *Pitta nympha* (Suppl. Table S1). A third data set used for reference mapping was a concatenation of 23 nuclear loci, primarily introns (evolving relatively slow), published for *Pitta guajana* and *Pitta erythrogaster* (Barker et al. 2002, Moyle et al. 2006, Hackett et al. 2008, Zuccon & Ericson 2012, see Suppl. Table S1). The fourth data set was a concatenation of 1,572 different UCE loci (500 kb nucleotides before filtering, see below) obtained by McCormack et al. (2013a) from the flanking regions of loci anchored by ultra-conserved elements (UCEs) distributed across the genome of the Banded Pitta *Pitta guajana*. These were downloaded from the Dryad data package McCormack et al. (2013b). Although we herein use the term "UCE" when

discussing this data set it should be borne in mind that the ultra-conserved elements *per se* are not part of our newly generated data set.

The reads were mapped against the different references using BWA mem v. 0.7.12 (Li & Durbin 2009). As expected the mapping of the mitogenomes resulted in the highest mapping coverage with a mean coverage of 998x (Suppl. Table S13). The data set of 23 nuclear genes (mostly introns) from other species of *Pitta* yielded a mean coverage of 188x. The mapping to the de-novo genome obtained in this study gave a lower coverage (mean 5.7x), as did the mapping of the UCEs (mean 3.5x).

5. *Variant calling*

The BAM-files produced when mapping the reads for each individual against the reference genome were merged using MergeSamFiles in Picard v. 1.118 (https://broadinstitute.github.io/picard). To convert the merged BAM file into genomic positions we first use mpileup in Samtools v. 0.1.9 (Li et al. 2009) and Samtools bcftools v. 1.1 (https://samtools.github.io/bcftools) to produce a BCF file to call genotypes and to list those found to be variant. In the last step low quality variants were filtered using Picard bcftools.

We called single-nucleotide polymorphisms (SNPs) for all individuals using the GATK v. 2.3.6 (McKenna et al. 2010) workflow that includes realign the merged BAM-file around indels using RealignerTargetCreator and IndelRealigner, detecting variants with UnifiedGenotyper and filtering high quality (>Q30) and low threshold (<Q4) variants using VariantFiltration. All variants that passed the filters were then used as a true training set for the VQSR (Variant Quality Score Recalibration) which was applied using ApplyRecalibration. Finally, SelectVariants was used to exclude non-variant and filtered loci.

Two sets of variants called by the Samtools and GATK workflows, respectively, were compared for concordance using GATK SelectVariants and VariantFiltration (the Samtools file was first cleaned from all positions that are not A, C, G or T in order to get GATK to work). From the file with concordance variants we removed 1) those variants where the distance to the adjacent SNP was 5 bp or less, 2) all non biallelic variants, and 3) those variants with a minor allele frequency (MAF) of 10% or less, or above 90%. After the filtering a total of 2,193,399 high quality variants were retained for analysis.

6. *Filtering alignments prior to the phylogenetic analyses*

The filtering procedure (using a custom designed workflow that is available at https://github.com/mozesblom.script) was performed in several steps and was identical for all three data sets. First, within each alignment the individual sequences were subject to check to filter out regions with large proportions of N’s. If more than half of the genotype calls within a window of 11 bp were N’s the entire window was replaced by N's. The window was then moved 5 bp and a new check was performed. The next step filters away all individuals with more than 40% N’s in the sequence. In the last step all columns in the alignment with more

than 40% N's are removed. This procedure substantially decreased the size of all the individual genes alignments (Suppl. Table S1). In the majority of cases this was mostly due to the deletion of columns with too large proportion of N's. In some cases was entire taxa removed from the alignment but for 14 of the 23 nuclear genes and for the mitochondrial genome all individuals were kept in the alignment (Suppl. Table S1). For four of the nuclear genes one individual was deleted from the alignment, and for three nuclear genes two individuals were removed.

7. *Phylogenetic analyses, dating and population genetic structure*

Best-fit maximum-likelihood trees were estimated individually for each of the 23 nuclear genes and the mitochondrial genome using RAxML v. 7.4.7 (Stamatakis 2014) applying the General Time Reversible model of nucleotide substitution. We also estimated best-fit maximum-likelihood trees for concatenated data sets consisting of all 23 nuclear genes for which taxon-complete alignments were available (totaling 24,072 bp), as well as for this data set combined with the mitochondrial data set (totaling 41,907 bp before filtering). Substitution models were selected using jModelTest v. 2.1.10 (Guindon & Gascuel 2003; Darriba et al. 2012) (Suppl. Table S1).

Based on the individual gene trees estimated for the 23 nuclear genes and the mitochondrial genome we estimated a species tree using MP-EST (Liu et al. 2010). Bootstrap values for the species tree were estimated in MP-EST after submitting 100 bootstrap trees obtained in the RAxML analysis for each gene.

We used the coalescent-based program SNAPP v. 1.3.0 in BEAST2 v. 2.4.8 (Bouckaert et al. 2014; Bryant et al. 2012) to perform a Bayesian MCMC analysis of the SNP data. The analyses may be interpreted with some caution as SNAPP assumes a strict isolation model with constant population sizes, a condition that is not met in several of the populations (see Results). To make the analysis computationally feasible we sampled 1% random SNPs from the original SNP data set. A total of 22,074 SNPs were used in the phylogenetic reconstruction of the western clade and 17,810 SNPs of the eastern clade. We chose wide and uninformative distributions as priors of the model parameters. The forward and backward mutation rates were set to be estimated during the course of the MCMC chain, and the rate parameters were sampled from an inverse gamma distribution. For the Yule prior for the species tree the lambda parameter, that governs the rate that species diverge, was uniformly distributed in the range of zero to one. After initial runs we found that the samples fell into two distinct and widely separated groups (corresponding to the western and eastern clades). These two groups were analysed separately to reduce the otherwise extremely long computational time and to enhance the graphic representation of the respective consensus tree. We run the analyses of the two clades for 561,000 iterations (eastern clade) and 72,500 iterations (western clade). We assessed convergence of the MCMC chains by plotting likelihood scores against iterations. We discarded as burn-in the first 56,000 runs from the analysis of the eastern clade and 327,900 from the analysis of the western clade. For each

clade we plotted the distribution of species trees in the posterior sample using DensiTree v. 2.1.11 (Bouckaert 2010).

We used 2,193,399 SNPs from genomic data to infer population structure and calculated the pairwise genetic distances among all samples to generate a neighbor-joining (NJ) tree using TreeBest v. 1.9.2 (Vilella et al. 2009). We performed a principal component analysis (PCA) using smartpca in EIGENSOFT v. 6.1.4 (Price et al. 2006). Population genetic structure was further inferred from the SNP data using the clustering algorithm FRAPPE v. 1.1 (Tang et al. 2005) and ADMIXTURE v. 1.3 (Alexander et al. 2009). To explore the divergence of the *Pitta* individuals, we set the pre-defined genetic clusters (K) from 2 to 9 to cover the maximum numbers of tentative lineages identified in the phylogenetic analysis of the mitochondrial genome (see Results). We run the analysis with 10,000 maximum iterations. We calculated the cross-validation error to choose what value for K that best explains the genetic grouping of the data (Alexander et al. 2009).

8. *Demographic history reconstruction*

The Generalized Phylogenetic Coalescent Sampler (G-PhoCS v. 1.2.3) (Gronau et al. 2011) was used to infer the demographic history of the "*Pitta sordida* species-complex", including population divergence times, ancestral population size, and migration rates. G-PhoCS is a coalescent-based analysis that assumes the input alignments to represent a set of putative "neutral loci" in which recombination occurred at negligible rates during the sample history. Herein we used data obtained by McCormack et al. (2013a) after employing simultaneous capture and high-throughput sequencing of hundreds of loci, anchored by ultra-conserved elements (UCEs). A total of 316 kb nucleotides obtained from the flanking regions of 1,572 different UCE loci distributed across the genome of the Banded Pitta *Pitta guajana* McCormack et al. (2013a). These were downloaded from the Dryad data package McCormack et al. (2013b) and used for reference mapping. We arbitrarily divided the 316 kb data set into 1,569 segments consisting of 202 bp each. The flanking regions around the UCEs are characterized by having a high variability and evolving neutrally (Edwards et al. 2017). We hypothesize that the UCE flanking regions used herein are reasonably independent of each other and thus appropriate for analysing with G-PhoCS. The program uses Markov Chain Monte Carlo to jointly sample model parameters and genealogies of the marker. G-PhoCS uses a predefined tree topology to estimate demographic parameters. We used the topology obtained in the maximum-likelihood analysis of the data set consisting of 23 nuclear genes and the mitochondrial genome combined. The only exception is that we pruned the taxon *palawanensis* from the tree after observing it made the initial results unstable. Possibly this is related to its uncertain phylogenetic position, which, as we will show below, may be because of a high level of introgression. As rough estimates of the initialization points (τ-initial) for divergence of the ancestral populations (deducted from the tree topology) we used the average *p*-distances within lineages for cytochrome *b*. The average genetic distance across all lineages was 0.0373 and we used this value as the mean α/β of our gamma prior for θ and set $\alpha = 2$, which resulted in a rather diffuse prior. We also set post-divergence migration

bands for certain geographically close populations to test if gene flow have occurred after they separated. More information about the G-PhoCS control file is given in Supplementary material. The Markov chains were run for 200,000 generations while sampling parameter values every 100th iteration. Burn-in and convergence of each run were determined with TRACER v. 1.5 (Drummond & Rambaut 2007). We repeated the G-PhoCS analysis with six separate runs to obtain reliable and stable estimates for the demographic parameters. We obtained posterior distributions of τ (coalescent branch lengths) and θ (ancestral scaled population sizes) for what we consider to be the best estimate of the species tree for the 28 samples of the "*Pitta sordida* species-complex". The parameters τ and θ were re-calculated to divergence times in units of years, effective population sizes, and migration rates by calibrating with the setting of generation time to 4.2 years (BirdLife International 2016) and neutral mutation rate to $4.6*10^{-9}$ (this mutation rate was obtained in a study of the collared flycatcher *Ficedula albicollis* [Smeds et al. 2016]). We defined four migration bands to estimate gene flow between nearby populations. The effective number of migrants per generation (2Nm) was calculated by multiplying the migration rate (m) estimated by G-PhoCS with the value of θ for the target population and divide by 4 (following Poelstra et al. 2018). Gene flow was calculated by multiplying the estimated per-generation rate with the number of generations that migration is allowed in the model (i.e. based on the time since the split between the populations).

The G-PhoCS analysis resulted in surprisingly young divergence times between some of the clades in the "*Pitta sordida* species-complex". As a comparison we also estimated divergence times using the hypothesis of a molecular clock for the mitochondrial data. We thus analysed a data set of mitochondrial genomes using Bayesian inference (10 million generations) in BEAST2 v. 2.4.8 (Drummond & Rambaut 2007; Bouckaert et al. 2014), applying the GTR + Γ model for nucleotide substitutions and assuming a Yule speciation process for the tree prior. The substitution model was selected using jModelTest v. 2.1.10 (Guindon & Gascuel 2003; Darriba et al. 2012). We applied a relaxed uncorrelated lognormal molecular clock to the data. As no fossils are available for this group of birds we used an evolutionary rate of 1.05% (± 0.35%) sequence divergence per lineage per million years to obtain absolute dates (Weir & Schluter 2007). The program Tracer (Rambaut & Drummond 2007) was used to assess convergence diagnostics.

9. *Genetic admixture between isolated island lineages*

We tested for differential gene flow between populations within the "*Pitta sordida* species-complex" by applying multi-population ABBA-BABA tests, using the abbababa2 function in ANGSD v. 0.921 (Soraggi et al. 2018). The ABBA-BABA statistic is based on the phylogeny (((P1; P2);P3);O) where A is the ancestral allele and B is the derived allele. ABBA-topologies are sites or loci at which the derived allele B is shared between the non-sister taxa P2 and P3, whereas P1 carries the ancestral allele, as defined by the outgroup O. In the BABA-topologies the derived allele B is instead shared by the non-sister taxa P1 and P3. It is predicted that the ABBA and BABA patterns should be equally frequent under the assumption of incomplete

lineage sorting without gene flow (Durand et al. 2011). An excess of ABBA or BABA allele patterns is thus indicative of gene flow between two of the non-sister taxa and can be detected using Patterson's D-statistic implemented in ANGSD (Korneliussen et al. 2014). We did separate analysis for the eastern and the western clades (see Results below), respectively, using an individual from the other clade as outgroup. The Z-scores were calculated by a block jack-knife approach where the whole genome was divided into 5 Mbp blocks (8692 blocks for clade 1 and 7915 for clade 2). The observed Z-scores were highly significant in almost all tests, also when the D-statistic approached zero. When interpreting the results we thus focused on the tests that yielded the largest D-statistic (we used 0.04 as cut-off value), i.e. where a significant introgression or hybridization was inferred.

We used the *f*4-statistic (Reich et al. 2009) to distinguish introgression from incomplete lineage sorting, based on allele frequencies of four populations (the description of the *f*4-statistics below builds largely on Meyer et al. [2017]). With populations A, B, C, and D, and the assumed population topology (A,B),(C,D), the *f*4-statistic is calculated as the product of the difference of allele frequencies between A and B, and between C and D. The *f*4-statistic is simply the mean of the *f*4 values of all individual SNPs. Under incomplete lineage sorting alone, the allele frequency differences between A and B should be independent of those between C and D, and the *f*4-statistic should be zero and thus indicate absence of introgression. However, if there is introgression between the two pairs of populations (e.g. A introgressed into C), this would lead to non-zero *f*4 values. We thus calculated the *f*4-statistic for sets of four taxa to test hypotheses of introgression (Suppl. Table S8). Introgression between one of the two species A and B and one of the species C and D is inferred if the *f*4-statistic is significantly different from zero (Reich et al. 2009). This is usually assessed based on standard errors calculated through a block jackknife procedure. The use of jackknife standard errors for confidence interval estimation assumes that the underlying data is normally distributed. However, this may often not be the case for the *f*4-statistic, especially with more divergent species-level allele frequency data. This is because with more divergent populations, a larger numbers of single nucleotide polymorphisms (SNPs) will be fixed within populations but different between them. As a result, the *f*4-statistic will be exactly zero for a large number of SNPs. If jackknife blocks include a large number of linked sites, the per-block *f*4-statistic may then also be close to zero more often than assumed under normality. To use the *f*4-statistic as a test of introgression with our population-level multimarker data set, we therefore applied not only a block jackknife procedure but also applied an approach to assess significance, which does not assume normality and accounts for linkage of genetic variation within markers. To this end, we conducted simulations to evaluate how often the observed *f*4-statistic can be reproduced in the absence of introgression, based on incomplete lineage sorting alone. The coalescent software fastsimcoal2 v.2.6 (Excoffier et al. 2013) was used to produce sequence data sets for four populations that are similar to the true data set in terms of size and amount of missing data. Simulation parameters for effective population sizes and divergence times were optimized during a burn-in phase. The burn-in phase was stopped as soon as parameter combinations were found with which the resulting simulated sequence

variation matched the observed sequence variation across all markers in the proportion of SNPs that are variable in more than one taxon and in the proportion of SNPs that are variable within both pairs of taxa. Subsequent to burn-in, 1000 sets of coalescent simulations were carried out, where in each of these sets sequence data was simulated separately for all markers included in a given four species comparison. We interpreted the observed *f*4-statistic as evidence for introgression if less than 5% of the 1000 data sets simulated without introgression produced *f*4 values at least as extreme as the observed.

Additionally, we also estimate the levels of inbreeding in isolated lineages within the "*Pitta sordida* species-complex" we estimated intra-individual runs of homozygosity (ROH), i.e. uninterrupted stretches of homozygous genotypes resulting from parents transmitting identical haplotypes to their offspring. In theory, inter-lineages gene flow could decrease homozygous level. For each individual we identified the number, size and distribution of large stretches of genome sequences (>250 kb) with none or very limited number of heterozygote sites. ROHs were identified on the genotypes of the 2,193,399 overlapping sites of each individual by using the *--homozyg* option of PLINK v. 1.9 (Purcell et al. 2007) and the following settings to call a segment as homozygote: a sliding window of 250 kbp, a minimum of 20 SNPs per window, a threshold of 0.05 for overlapping homozygote windows, and a maximum of 15 missing sites and three heterozygote positions.

The degree to which genetic differentiation can be explained by the geographic distance between sampling localities was assessed by plotting the genomic distance (*p*-distances) between each pair of individuals against their geographic distance.

10. Demographic fluctuations of genetic lineages

We used PopSizeABC (Boitard et al. 2016), an approximate Bayesian computation pipeline, to estimate temporal variation in effective population size (N_e) for individuals in each lineage. If a population has gene flow with another population, e.g. through migration, we may expect a demographic fluctuation in these populations over the time. PopSizeABC uses the SNP data set (only SNPs with a minor allele count above two) to calculate summary statistics of the genome-wide allele frequency spectrum (AFS) and the average zygotic linkage disequilibrium (LD) at specific time bins (Boitard et al. 2016). These statistics are first calculated for an empirical data set and then compared with the corresponding statistics calculated from a large number of simulated data sets. The simulated data sets are obtained by cutting the empirical data set into segments of 2 million bp each and then randomly select 100 such segments. N_e was estimated in 21 discrete time windows between 2,400 to 130,000 years BP. In the analyses we set the generation time to 4.2 years (BirdLife International 2016), the recombination rate to $1.0*10^{-8}$, and the genomic mutation rate per generation to $4.6*10^{-9}$ (Smeds et al. 2016). We compared the summary statistics for the empirical data sets with 400,000 simulated data sets to identify the simulations that are most similar. These were then selected by applying a simple rejection method with an acceptance (tolerance) rate of 0.001.

In addition, we used DnaSP v. 6.11.01 (Rozas et al. 2017) to test the model of demographic expansion of different lineages within the "*Pitta sordida* species-complex" by comparing observed and expected distributions of differences between pairs of haplotypes and applying two neutrality tests, the raggedness index r_g (Harpending et al. 1993) and R_2 (which is particularly suitable for small sample sizes, Ramos-Onsins and Rozas 2002). Under the assumption of a recent expansion a unimodal mismatch distribution is expected, while in populations at demographic equilibrium the distribution is expected to be highly multimodal. Probability values for the neutrality tests were obtained by coalescent simulations with 10,000 replicates. Statistical significance of r_g and R_2 thus indicates that the studied population is in equilibrium. A non-significant statistics thus indicates that there is no support for a stable population, but it should be noted that it does not provide absolute evidence for an expanding population either. Multiple peaks may appear in the mismatch distribution also when the data is substructured. Furthermore, in case of multispecies phylogenies as herein a signal for population expansion may be translated to rapid diversification of lineages or speciation events.

REFERENCES

Alexander, D.H., Novembre, J. & Lange, K. 2009. Fast model-based estimation of ancestry in unrelated individuals. Genome Research, 19: 1655-1664.

Andrews, S. 2010. FastQC: a quality control tool for high throughput sequence data. Available online at: http://www.bioinformatics.babraham.ac.uk/projects/fastqc

Barker, F.K., Barrowclough, G.F. & Groth, J.G. 2002. A phylogenetic hypothesis for passerine birds: taxonomic and biogeographic implications of an analysis of nuclear DNA sequence data. Proceedings of the Royal Society London, Series B, 269: 295-308.

BirdLife International 2016. *Pitta sordida. The IUCN Red List of Threatened Species* 2016: e.T103656903A93693998. http://dx.doi.org/10.2305/IUCN.UK.2016-3.RLTS.T103656903A93693998.en. Downloaded on 23 February 2019.

Boitard, S., Rodríguez,W., Jay, F., Mona, S. & Austerlitz, F. 2016. Inferring population size history from large samples of genome-wide molecular data - an approximate Bayesian computation approach. PLoS Genetics, 12(3): e1005877.

Bolger, A.M., Lohse, M. & Usadel, B. 2014. Trimmomatic: a flexible trimmer for Illumina sequence data. Bioinformatics, 30: 2114-2120.

Bouckaert, R., Heled, J., Kühnert, D., Vaughan, T., Wu, C-H., Xie, D., Suchard, M.A., Rambaut, A., & Drummond, A.J. 2014. BEAST 2: A Software Platform for Bayesian Evolutionary Analysis. PLoS Computational Biology, 10(4): e1003537.

Bouckaert, R.R. 2010. DensiTree: making sense of sets of phylogenetic trees. Bioinformatics, 26: 1372-1373.

Bryant, D., Bouckaert, R., Felsenstein, J., Rosenberg, N. & RoyChoudhury, A. 2012. Inferring species trees directly from biallelic genetic markers: bypassing gene trees in a full coalescent analysis. Molecular Biology and Evolution, 29: 1917-1932.

Darriba, D., Taboada, G.L., Doallo, R. & Posada, D. 2012. jModelTest 2: more models, new heuristics and parallel computing. Nature Methods, 9: 772.

del Hoyo, J., Collar, N. & Kirwan, G.M. 2018a. Biak Hooded Pitta (*Pitta rosenbergii*). In del Hoyo, J., Elliott, A., Sargatal, J., Christie, D.A. & de Juana, E. (eds.). *Handbook of the Birds of the World Alive.* Lynx Edicions, Barcelona. (retrieved from https://www.hbw.com/node/1343577 on 29 April 2018).

del Hoyo, J., Collar, N. & Kirwan, G.M. 2018b. Eastern Hooded Pitta (*Pitta novaeguineae*). In del Hoyo, J., Elliott, A., Sargatal, J., Christie, D.A. & de Juana, E. (eds.). *Handbook of the Birds of the World Alive.* Lynx Edicions, Barcelona. (retrieved from https://www.hbw.com/node/1343576 on 29 April 2018).

Drummond, A.J. & Rambaut, A. 2007. BEAST: Bayesian evolutionary analysis by sampling trees. BMC Evolutionary Biology, 7: 214.

Durand, E.Y., Patterson, N., Reich, D. & Slatkin, M. 2011. Testing for ancient admixture between closely related populations. Molecular Biology and Evolution, 28: 2239-2252.

Edwards, S.V., Cloutier, A. & Baker, A.J. 2017. Conserved nonexonic elements: a novel class of marker for phylogenomics. Systematic Biology, 66: 1028-1044.

Erritzoe, J. & Erritzoe, H.B. 1998. *Pittas of the World: A Monograph on the Pitta Family.* Lutterworth Press, Cambridge, UK.

Erritzoe, J. & Kirwan, G.M. 2018. Western Hooded Pitta (*Pitta sordida*). In del Hoyo, J., Elliott, A., Sargatal, J., Christie, D.A. & de Juana, E. (eds.). *Handbook of the Birds of the World Alive.* Lynx Edicions, Barcelona. (retrieved from https://www.hbw.com/node/57570 on 29 April 2018).

Erritzoe, J. 2003. Family Pittidae (pittas). Pp. 106–160 in del Hoyo, J., Elliott, A. & Christie D.A. (eds.) *Handbook of the Birds of the World*, vol. 8. Lynx Edicions, Barcelona.

Excoffier, L., Dupanloup, I., Huerta-Sachez, E., Sousa, V.C. & Foll, M. 2013. Robust demographic inference from genomic and SNP data. PLoS Genetics, 9:e1003905.

Gronau, I., Hubisz, M.J., Gulkom B., Danko, C.G. & Siepel, A. 2011. Bayesian inference of ancient human demography from individual genome sequences. Nature Genetics, 43: 1031-1034.

Guindon, S. & Gascuel, O. 2003. A simple, fast and accurate method to estimate large phylogenies by maximum-likelihood". Systematic Biology, 52: 696-704.

Hackett, S.J., Kimball, R.T., Reddy, S., Bowie, R.C.K., Braun, E.L., Braun, M.J., Chojnowski, J.L., Cox, W.A., Han, K.-L., Harshman, J., Huddleston, C., Marks, B.D., Miglia, K.J., Moore, W.S., Sheldon, F.H., Steadman, D.W., Witt, C.C. & Yuri, T. 2008. A phylogenomic study of birds reveals their evolutionary history. Science, 320: 1763-1768.

Harpending, H.C., Sherry, S.T., Rogers, A.R. & Stoneking, M. 1993. Genetic structure of ancient human populations. Current Anthropology, 34: 483-496.

Korneliussen, T.S., Albrechtsen, A. & Nielsen, R. 2014. ANGSD: Analysis of Next Generation Sequencing Data. BMC Bioinformatics, 15: 356.

Li, H. & Durbin, R. 2009. Fast and accurate short read alignment with Burrows-Wheeler transform. Bioinformatics, 25: 1754-1760.

Li, H., Handsaker, B., Wysoker, A., Fennell, T., Ruan, J., Homer, N., Marth, G., Abecasis, G., Durbin, R. 2009. The Sequence Alignment/Map format and SAMtools. Bioinformatics, 25: 2078-2079. doi: 10.1093/bioinformatics/btp352

Liu, L., Yu, L. & Edwards, S.V. 2010. A maximum pseudo-likelihood approach for estimating species trees under the coalescent model. BMC Evolutionary Biology, 10: 302.

Mayr, E., 1979. Family Pittidae. In Traylor, M.A. Jr. (ed.), Check-list of Birds of the World 8: i-xv, 1-365. Cambridge, Mass., Mus. Comp. Zool. Pp. 310-329.

McCormack, J.E., Harvey, M.G., Faircloth, B.C., Crawford, N.G., Glenn, T.C. & Brumfield, R.T. 2013a. Data from: A phylogeny of birds based on over 1,500 loci collected by target enrichment and high-throughput sequencing. Dryad Digital Repository. https://doi.org/10.5061/dryad.sd080

McCormack, J.E., Harvey, M.G., Faircloth, B.C., Crawford, N.G., Glenn, T.C. & Brumfield, R.T. 2013b. A phylogeny of birds based on over 1,500 loci collected by target enrichment and high-throughput sequencing. PLoS ONE 8(1): e54848.

McKenna, A., Hanna, M., Banks, E., Sivachenko, A., Cibulskis, K., Kernytsky, A., Garimella, K., Altshuler, D., Gabriel, S., Daly, M. & DePristo, M.A. 2010. The Genome Analysis Toolkit: a MapReduce framework for analyzing next-generation DNA sequencing data. Genome Res., 20: 1297-1303.

Meyer, B.S., Matschiner, M. & Salzburger, W. 2017. Disentangling incomplete lineage sorting and introgression to refine species-tree estimates for Lake Tanganyika cichlid fishes. Systematic Biology, 66: 531-550.

Meyer, M. & Kircher, M. 2010. Illumina sequencing library preparation for highly multiplexed target capture and sequencing. Cold Spring Harbor Protocols, 6: pdb. prot5448.

Moyle, R.G., Chesser, R.T., Prum, R.O., Schikler, P. & Cracraft, J. 2006. Phylogeny and evolutionary history of Old World suboscine birds (Aves: Eurylaimides). American Museum Novitates, 3544: 1-22.

Petersen, K.R., Street, D.A., Gerritsen, A.T., Hunter, S.S. & Settles, M.L. 2015. Super deduper, fast PCR duplicate detection in fastq files. Proceedings of the 6th ACM Conference on Bioinformatics, Computational Biology and Health Informatics, pp. 491-492. DOI: 10.1145/2808719.2811568

Poelstra, J.W., Richards, E.J. & Martin, C.H. 2018. Speciation in sympatry with ongoing secondary gene flow and a potential olfactory trigger in a radiation of Cameroon cichlids. Molecular Ecology, 27: 4270-4288.

Price, A.L., Patterson, N.J., Plenge, R.M., Weinblatt, M.E., Shadick, N.A. & Reich, D. 2006. Principal components analysis corrects for stratification in genome-wide association studies. Nature Genetics, 38: 904-909.

Purcell, S., Neale, B., Todd-Brown, K., Thomas, L., Ferreira, M.A.R., Bender, D., Maller, J., Sklar, P., de Bakker, P.I.W., Daly, M.J. & Sham, P.C. 2007. PLINK: a toolset for whole-genome association and population-based linkage analysis. American Journal of Human Genetics, 81: 559-575.

Rambaut, A., Drummond, A.J., Xie, D., Baele, G. & Suchard, M.A. 2018. Posterior summarisation in Bayesian phylogenetics using Tracer 1.7. Systematic Biology, 67: 901-904.

Ramos-Onsins, S.E. & Rozas, J. 2002. Statistical properties of new neutrality tests against population growth. Molecular Biology and Evolution, 19: 2092-2100.

Reich, D., Thangaraj, K., Patterson, N., Price, A.L. & Singh, L. 2009. Reconstructing Indian population history. Nature, 461: 489-494.

Rozas, J., Ferrer-Mata, A., Sánchez-DelBarri, J.C., Guirao-Rico, S., Librado, P., Ramos-Onsins, S.E. & Sánchez-Gracia, A. 2017. DnaSP v6: DNA Sequence Polymorphism Analysis of Large Datasets. Molecular Biology and Evolution, 34: 3299-3302.

Smeds, L., Qvarnström, A. & Ellegren, H. 2016. Direct estimate of the rate of germline mutation in a bird. Genome Research, 26: 1211-1218.

Soraggi, S., Wiuf, C. & Albrechtsen, A. 2018. Powerful Inference with the D-Statistic on Low-Coverage Whole-Genome Data. G3, 8: 551-566. doi: 10.1534/g3.117.300192.

Stamatakis, A. 2014. RAxML version 8: a tool for phylogenetic analysis and post-analysis of large phylogenies. Bioinformatics, 30: 1312-1313.

Tang, H., Peng, J., Wang, P. & Risch, N. 2005. Estimation of individual admixture: analytical and study design considerations. Genetic Epidemiology, 28: 289-301.

Weir, J.T. & Schluter, D. 2007. The latitudinal gradient in recent speciation and extinction rates of birds and mammals. Science, 315: 1574-1576.

Vilella, A.J., Severin, J., Ureta-Vidal, A., Heng, L., Durbin, R., Birney, E. 2009. EnsemblCompara GeneTrees: Complete, duplication-aware phylogenetic trees in vertebrates. Genome Research, 19: 327-335.

Zhang, G.J., Li, C., Li, Q.Y., Li, B., Larkin, D.M., Lee, C., Storz, J.F., Antunes, A., Greenwold, M.J., Meredith, R.W., Ödeen, A., Cui, J., Zhou, Q., Xu, L.H., Pan, H.L., Wang, Z.J., Jin, L.J., Zhang, P., Hu, H.F., Yang, W., Hu, J., Xiao, J., Yang, Z.K., Liu, Y., Xie, Q.L., Yu, H., Lian, J.M., Wen, P., Zhang, F., Li, H., Zeng, Y.L., Xiong, Z.J., Liu, S.P., Zhou, L., Huang, Z.Y., An, N., Wang, J., Zheng, Q.M., Xiong, Y.Q., Wang, G.B., Wang, B., Wang, J.J., Fan, Y., Da Fonseca, R.R., Alfaro-Nunez, A., Schubert, M., Orlando, L., Mourier, T., Howard, J.T., Ganapathy, G., Pfenning, A., Whitney, O., Rivas, M.V., Hara, E., Smith, J., Farre, M., Narayan, J., Slavov, G., Romanov, M.N., Borges, R., Machado, J.P., Khan, I., Springer, M.S., Gatesy, J., Hoffmann, F.G., Opazo, J.C., Håstad, O., Sawyer, R.H., Kim, H., Kim, K.W., Kim, H.J., Cho, S., Li, N., Huang, Y.H., Bruford, M.W., Zhan, X.J., Dixon, A., Bertelsen, M.F., Derryberry, E., Warren, W., Wilson, R.K., Li, S.B., Ray, D.A., Green, R.E., O'brien, S.J., Griffin, D., Johnson, W.E., Haussler, D., Ryder, O.A., Willerslev, E., Graves, G.R., Alstrom, P., Fjeldså, J., Mindell, D.P., Edwards, S.V., Braun, E.L., Rahbek, C., Burt, D.W., Houde, P., Zhang, Y., Yang, H.M., Wang, J., Jarvis, E.D., Gilbert, M.T.P., Wang, J., Consortium AG. 2014. Comparative genomics reveals insights into avian genome evolution and adaptation. Science, 346: 1311-1320.

Zhang, J., Kobert, K., Flouri, T. & Stamatakis, A. 2014. PEAR: a fast and accurate Illumina Paired-End reAd mergeR. Bioinformatics, 30: 614-620.

Zuccon, D. & Ericson, P.G.P. 2012. Molecular and morphological evidences place the extinct New Zealand endemic *Turnagra capensis* in the Oriolidae. Molecular Phylogenetics and Evolution, 62: 414-426.
