# Supplementary material for: Genomic differentiation tracks earth-historic isolation in an Indo-Australasian archipelagic pitta (Pittidae; Aves) complex
Source: BMC Evol Biol. 2019 Jul 24;19:151. doi: 10.1186/s12862-019-1481-5 (PMC6657069; doi:10.1186/s12862-019-1481-5)
Supplement: Supplementary file 2 — Supplementary tables and figures. Table S1 Data sets used for reference mapping. Table S2 Comparison of different tree topologies reconstructed in the phylogenetic analyses of different data sets and by employing different methods. Note that several topologies are incompatible with each other. Table S3 Calculated effective population sizes (Ne) inferred by G-PhoCS using the predefined tree topology in Fig. 2 (after pruning palawanensis from the tree, see text in Additional file 1 for details) and after calibrating the estimated parameter θ assuming an average mutation rate of 4.6*10− 9substitutions per generation and an average generation length of 4.2 year. Table S4 Calculated divergence times (T) inferred by G-PhoCS using the predefined tree topology in Fig. 2 (after pruning palawanensis from the tree, see text in Additional file 1 for details) and after calibrating the estimated parameter τ assuming an average mutation rate of 4.6*10− 9 substitutions per generation and an average generation length of 4.2 year. Table S5 Estimates of mean number of migrants per generation (Ms-t) for the migration bands inferred by bands inferred by G-PhoCS using the predefined tree topology in Fig. 2 (after pruning palawanensis from the tree, see text in Additional file 1 for details). Table S6 Comparisons of estimates of divergence times for major clades within the “Pitta sordida species-complex” using a molecular clock approach in Beast2 and genomic mutation rates in G-PhoCS. In the G-PhoCS analysis we used two different estimates of the mutation rate in passerines; 3.0*10− 9(Zhang et al. 2014) and 4.6*10− 9(Smedset al. 2016). Table S7 Individuals and scaffolds in which longer (> 250 Mb) stretches of homozygosity was observed. Table S8 The f4-statistic was calculated in four-taxon comparisons to test different hypotheses of introgression. Table S9 Analysis of mismatch distribution in populations within the “Pitta sordida species-complex” from which two or more individuals were sa [file 12862_2019_1481_MOESM2_ESM.pdf]

BMC Evolutionary Biology

Genomic differentiation tracks earth-historic isolation in an Indo-Australasian archipelagic pitta (Pittidae; Aves) complex

Per G.P. Ericson, Yanhua Qu, Pamela C. Rasmussen, Mozes P.K. Blom, Frank E. Rheindt & Martin Irestedt

Additional file 2: Supplementary tables and figures.

Legends

Table S1: Data sets used for reference mapping.

Table S2: Comparison of different tree topologies reconstructed in the phylogenetic analyses of different data sets and by employing different methods. Note that several topologies are incompatible with each other.

Table S3: Calculated effective population sizes (N_e) inferred by G-PhoCS using the predefined tree topology in Figure 2 (after pruning *palawanensis* from the tree, see text in Additional file 1 for details) and after calibrating the estimated parameter θ assuming an average mutation rate of $4.6*10^{-9}$ substitutions per generation and an average generation length of 4.2 year.

Table S4: Calculated divergence times (T) inferred by G-PhoCS using the predefined tree topology in Figure 2 (after pruning *palawanensis* from the tree, see text in Additional file 1 for details) and after calibrating the estimated parameter τ assuming an average mutation rate of $4.6*10^{-9}$ substitutions per generation and an average generation length of 4.2 year.

Table S5: Estimates of mean number of migrants per generation ($M_{s\text{-}t}$) for the migration bands inferred by G-PhoCS using the predefined tree topology in Figure 2 (after pruning *palawanensis* from the tree, see text in Additional file 1 for details).

Table S6: Comparisons of estimates of divergence times for major clades within the "Pitta sordida species-complex" using a molecular clock approach in Beast2 and genomic mutation rates in G-PhoCS. In the G-PhoCS analysis we used two different estimates of the mutation rate in passerines; $3.0*10^{-9}$ (Zhang et al. 2014) and $4.6*10^{-9}$ (Smeds et al. 2016).

Table S7: Individuals and scaffolds in which longer (>250 Mb) stretches of homozygosity was observed.

Table S8: The *f4*-statistic was calculated in four-taxon comparisons to test different hypotheses of introgression. For each comparison, the number of biallelic SNPs used for the test and the percentage of SNPs that are variable in both pairs of sister species are given. Significance of *f4* values was assessed with a block Jackknife procedure and with ILS-based simulations.

Table S9: Analysis of mismatch distribution in populations within the "*Pitta sordida* species-complex" from which two or more individuals were sampled. The raggedness indices R_g (Harpending et al. 1993) and R_2 (Ramos-Onsins and Rozas 2002) evaluates departure from the null-hypothesis that the population has been expanding. ns = not significant, * $p < 0.05$, ** $p < 0.01$.

Table S10: Admixture signatures from D-statistics (ABBA-BABA test). Non-sister taxa with signatures of considerable gene flow (D-statistic higher than 0.05 or lower than -0.05) are marked in bold.

Table S11: Alternative taxonomic classifications of the taxa included in the "*Pitta sordida* species-complex".

Table S12: Specimen data for the samples used in the study. Acronyms: AMNH, American Museum of Natural History; ZMA, Zoological Museum Amsterdam (Naturalis, Leiden); LKCNHM, Lee Kong Chian Natural History Museum Singapore.

Table S13: Mapping coverage estimated with *genomecov* in BEDTools v.2.26.0.

Fig. S1: Phylogenetic relationships within the "Pitta sordida" species-complex reconstructed with a neighbor-joining analysis of 2,193,399 high-quality SNPs. Numbers at the nodes are bootstrap values (after 100 replicates).

Fig. S2: Phylogenetic relationships within the "*Pitta sordida*" species-complex inferred with MP-EST. Bootstrap values were estimated in MP-EST after submitting 100 bootstrap trees obtained in the RAxML analysis for each of 23 nuclear genes.

Fig. S3: Phylogenetic relationships within the "*Pitta sordida*" species-complex inferred with MP-EST. Bootstrap values were estimated in MP-EST after submitting 100 bootstrap trees obtained in the RAxML analysis for each of 23 nuclear genes plus the mitochondrial genome.

Fig. S4: Posterior distribution of trees obtained in a Bayesian analysis using SNAPP based on 22,074 biallelic SNPs shared across the taxa in the western clade. Higher density areas of the tree indicate greater topology agreement.

Fig. S5: Posterior distribution of trees obtained in a Bayesian analysis using SNAPP based on 17,810 biallelic SNPs shared across the taxa in the eastern clade. Higher density areas of the tree indicate greater topology agreement.

Fig. S6: Principal component analysis (PCA) of the genomic variation among the samples assigned to the taxa *mulleri*, *cucullata* and *bangkana*. Only the first two components, together representing 37% of the total variation, are shown.

Fig. S7: Divergence times and effective population sizes (Ne) inferred by G-PhoCS using the predefined tree topology from Figure 2 (after pruning *palawanensis*, see Additional file 1). Estimated divergence times and effective population sizes are calibrated assuming an average mutation rate of $4.6*10^{-9}$ substitutions per generation and an average generation time of 4.2 year. See Additional file 2, Tables S3-S5 for details.

Fig. S8: D-statistic (ABBA-BABA) for the tested comparisons within the western clade. The red squares indicate D-statistic values for the complete data set. The tested phylogeny is formatted as (((P1,P2),P3),P4), where P4 is outgroup. Non-sister taxa with signatures of considerable gene flow (absolute D value > ± 0.05) are marked in bold.

Fig. S9: D-statistic (ABBA-BABA) for the tested comparisons within the eastern clade. The red squares indicate D-statistic values for the complete data set. The tested phylogeny is formatted as (((P1,P2),P3),P4), where P4 is outgroup. Non-sister taxa with signatures of considerable gene flow (absolute D value > ± 0.05) are marked in bold.

Fig. S10: Cumulative size of the genome fraction allocated in runs of homozygosity (>250 kb) per sample.

Fig. S11: Cumulative size of the genome fraction allocated in runs of homozygosity (>250 kb) plotted against the size of the island from where the sample was collected.

Fig. S12: Variation in effective size (N_e) in populations represented by more than one individual inferred with the approximate Bayesian computation pipeline PopSizeABC using 1,304,779 SNPs. Summary statistics of the genome-wide allele frequency spectrum (AFS) and the average zygotic linkage disequilibrium (LD) are first calculated at 21 discrete time windows, between 2,400 to 130,000 years BP, based on the empirical data set. These statistics are then compared with the corresponding statistics calculated from 400,000 simulated data sets. In the analyses was used a generation time of 4.2 year, a recombination rate of $1.0*10^{-8}$, and a genomic mutation rate per generation of $4.6*10^{-9}$.

Fig. S13: Scatterplot of the genetic (*p*-distances) and geographic distances (km) between all pairs of samples in the western clade. Pearson's correlation coefficient r and the trend line (calculated by linear regression) that best models the data are shown in the diagram.

Fig. S14: Scatterplot of the genetic (*p*-distances) and geographic distances (km) between all pairs of samples in the eastern clade. Pearson's correlation coefficient r and the trend line (calculated by linear regression) that best models the data are shown in the diagram.

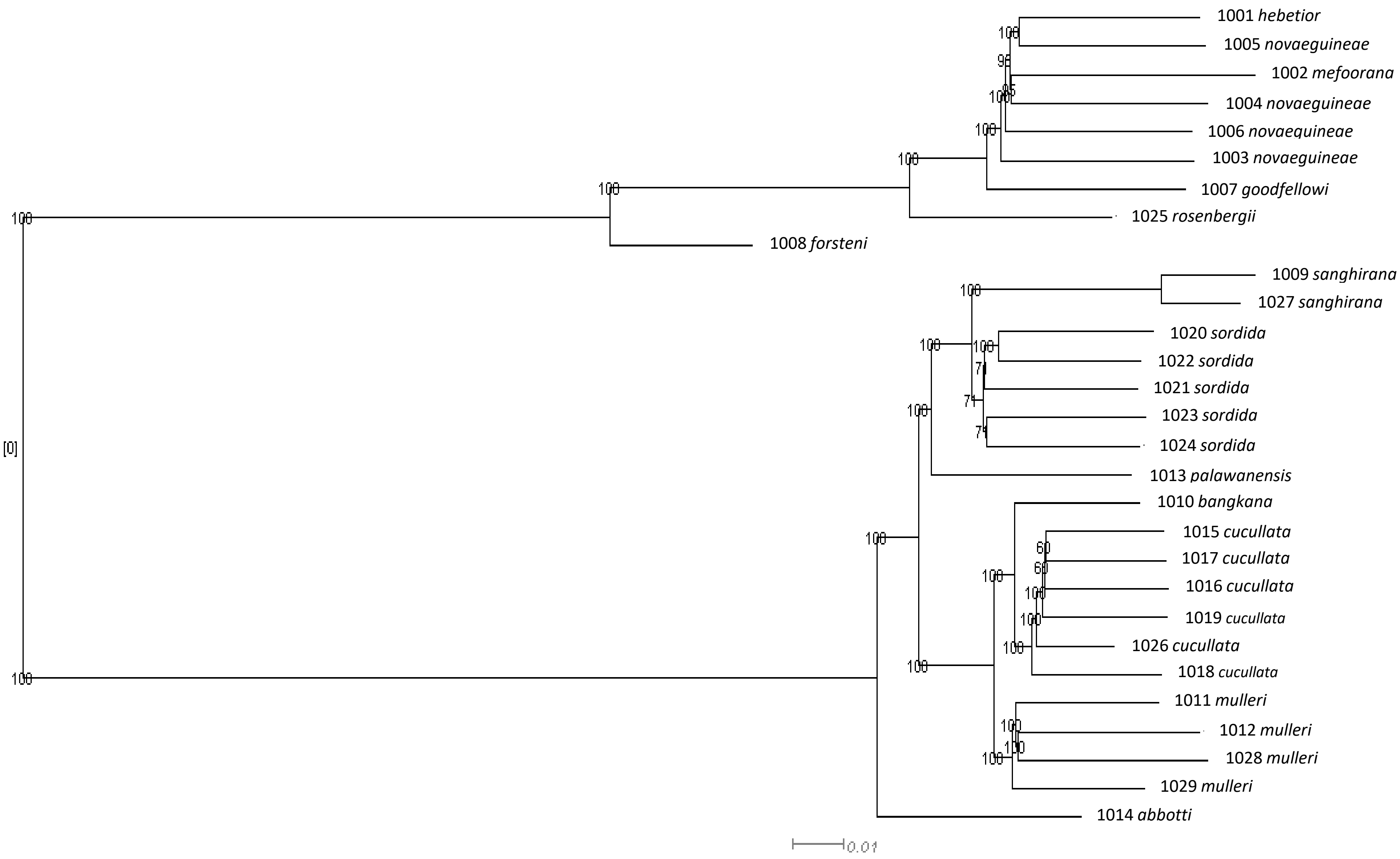

Fig. S1: Phylogenetic relationships within the "*Pitta sordida*" species-complex reconstructed with a neighbor-joining analysis of 2,193,399 high-quality SNPs. Numbers at the nodes are bootstrap values (after 100 replicates).

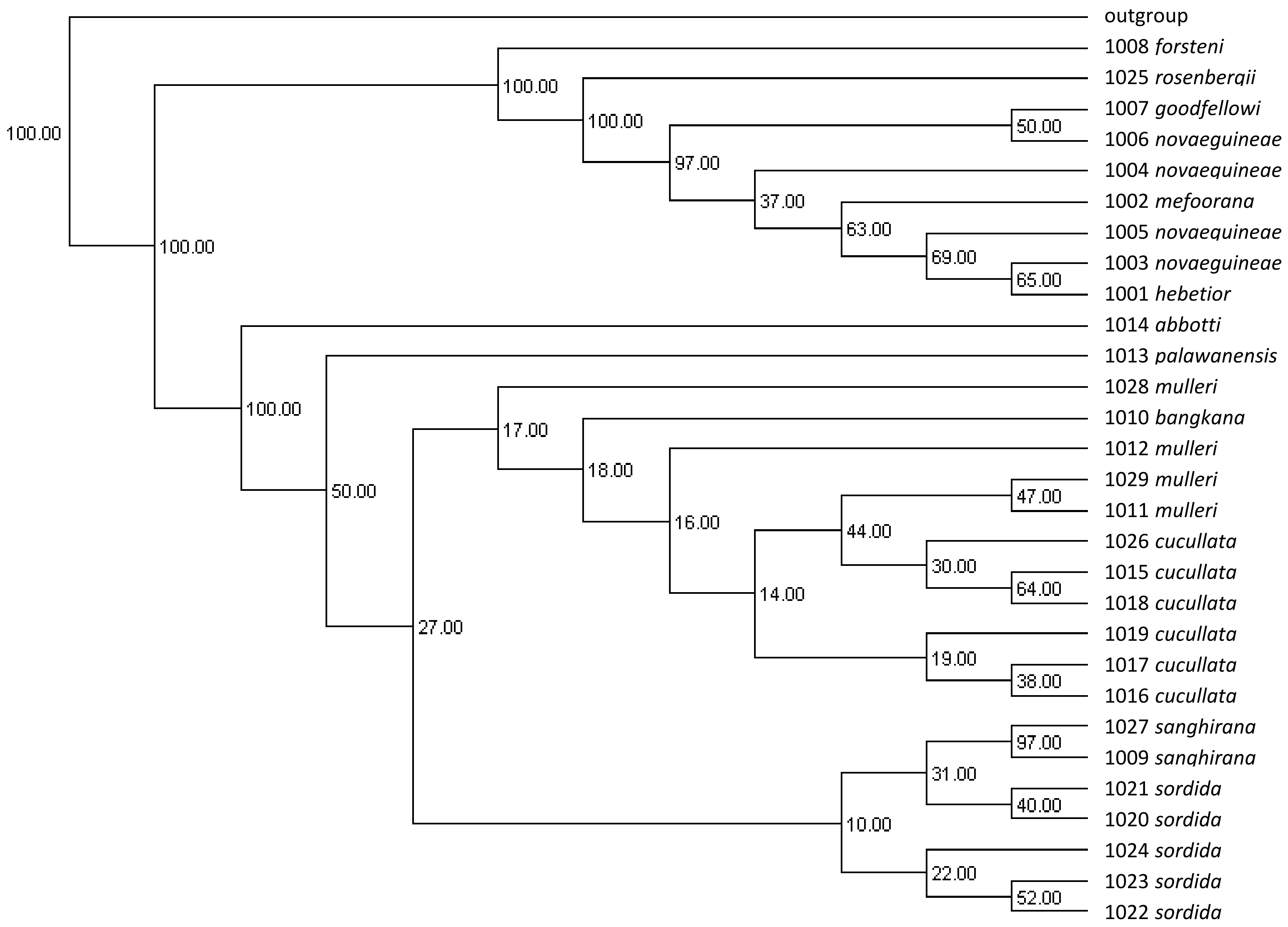

Fig. S2: Phylogenetic relationships within the "*Pitta sordida*" species-complex inferred with MP-EST. Bootstrap values were estimated in MP-EST after submitting 100 bootstrap trees obtained in the RAxML analysis for each of 23 nuclear genes.

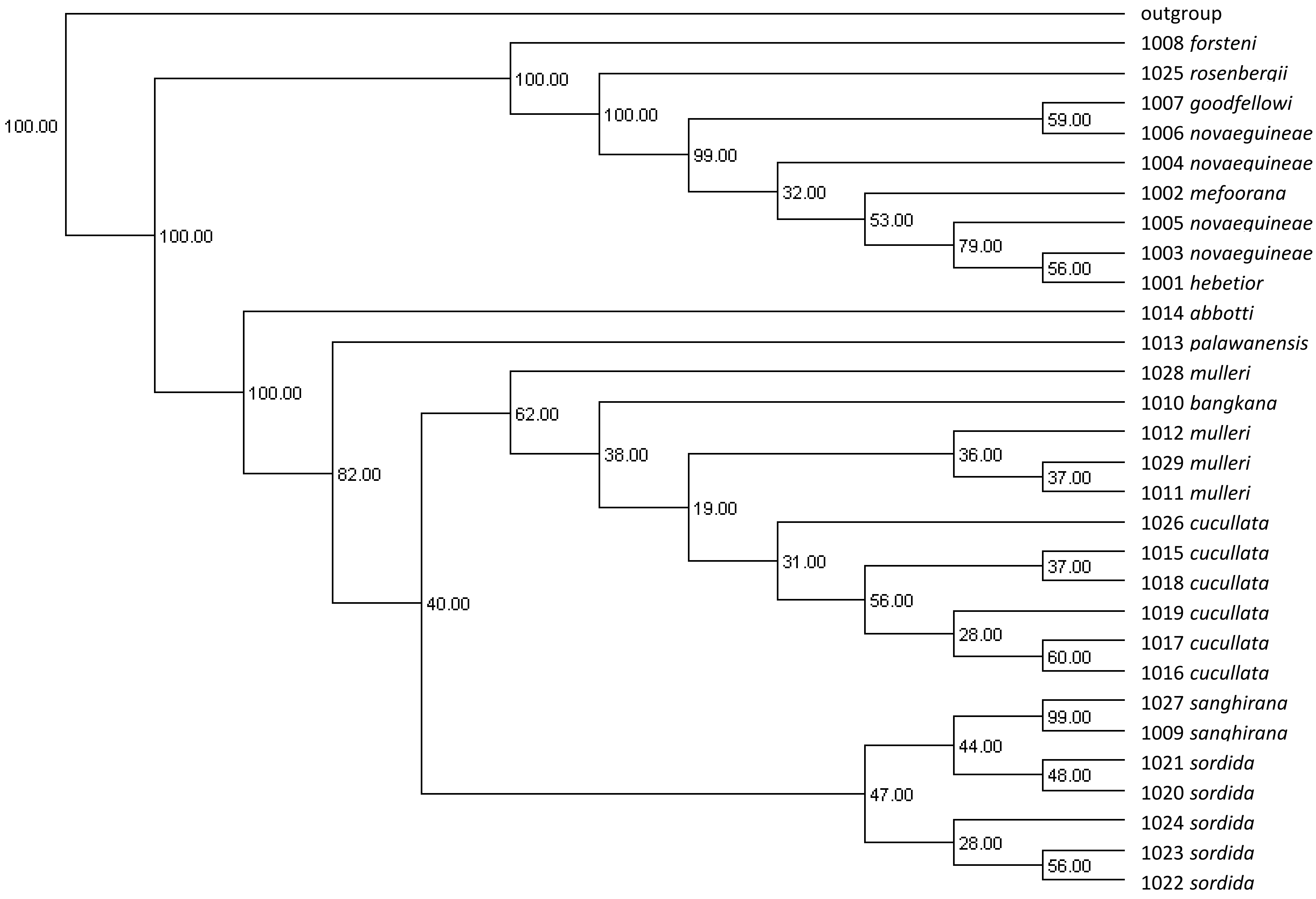

Fig. S3: Phylogenetic relationships within the "*Pitta sordida*" species-complex inferred with MP-EST. Bootstrap values were estimated in MP-EST after submitting 100 bootstrap trees obtained in the RAxML analysis for each of 23 nuclear genes plus the mitochondrial genome.

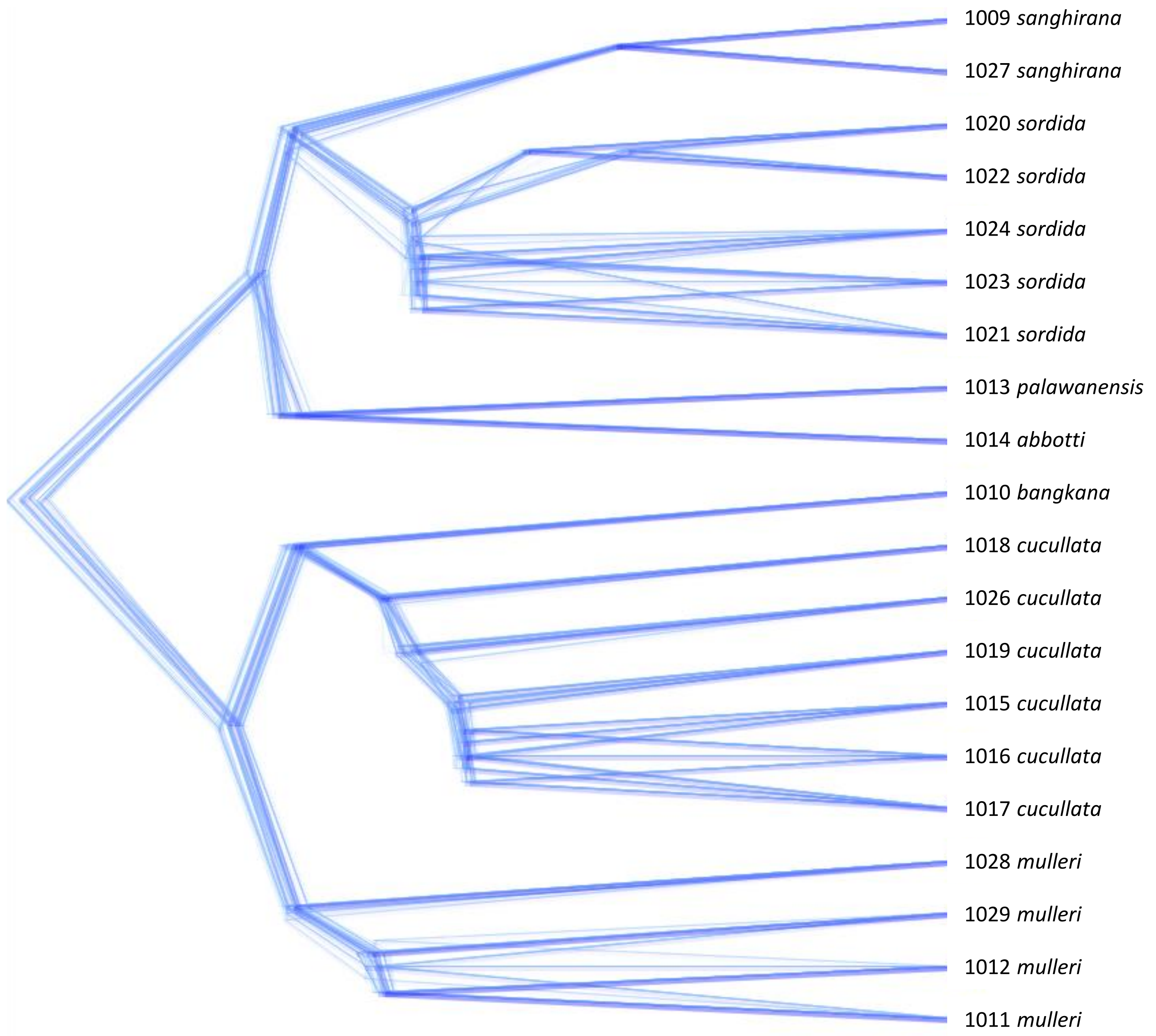

Fig. S4: Posterior distribution of trees obtained in a Bayesian analysis using SNAPP based on 22,074 biallelic SNPs shared across the taxa in the western clade. Higher density areas of the tree indicate greater topology agreement.

Pitta sordida ssp. – SNAPP tree eastern clade

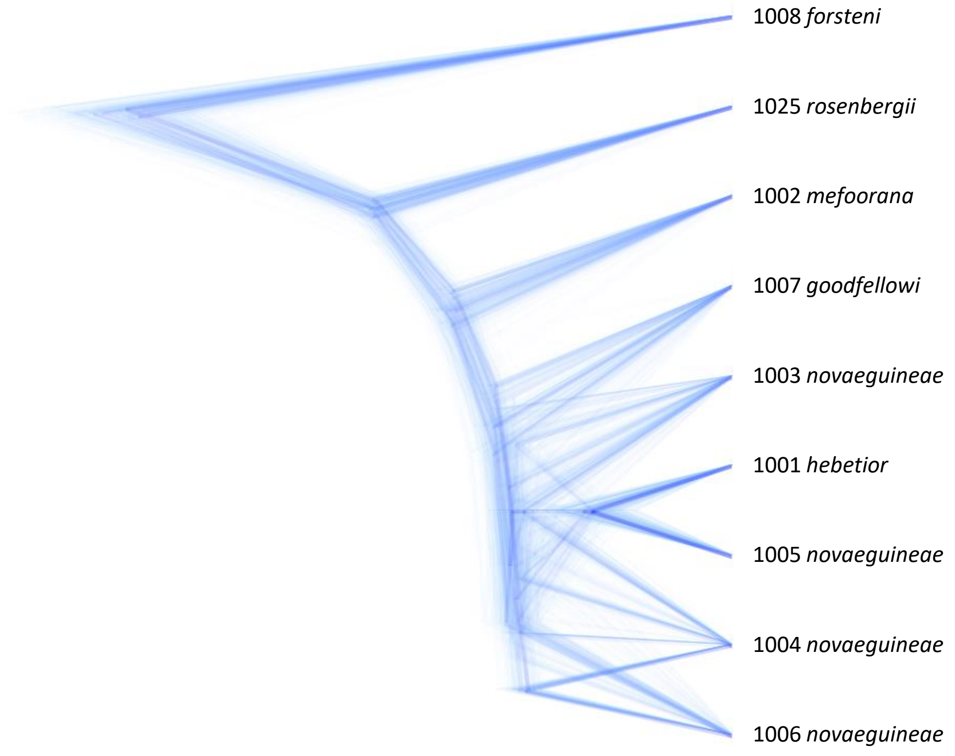

Fig. S5: Posterior distribution of trees obtained in a Bayesian analysis using SNAPP based on 17,810 biallelic SNPs shared across the taxa in the eastern clade. Higher density areas of the tree indicate greater topology agreement.

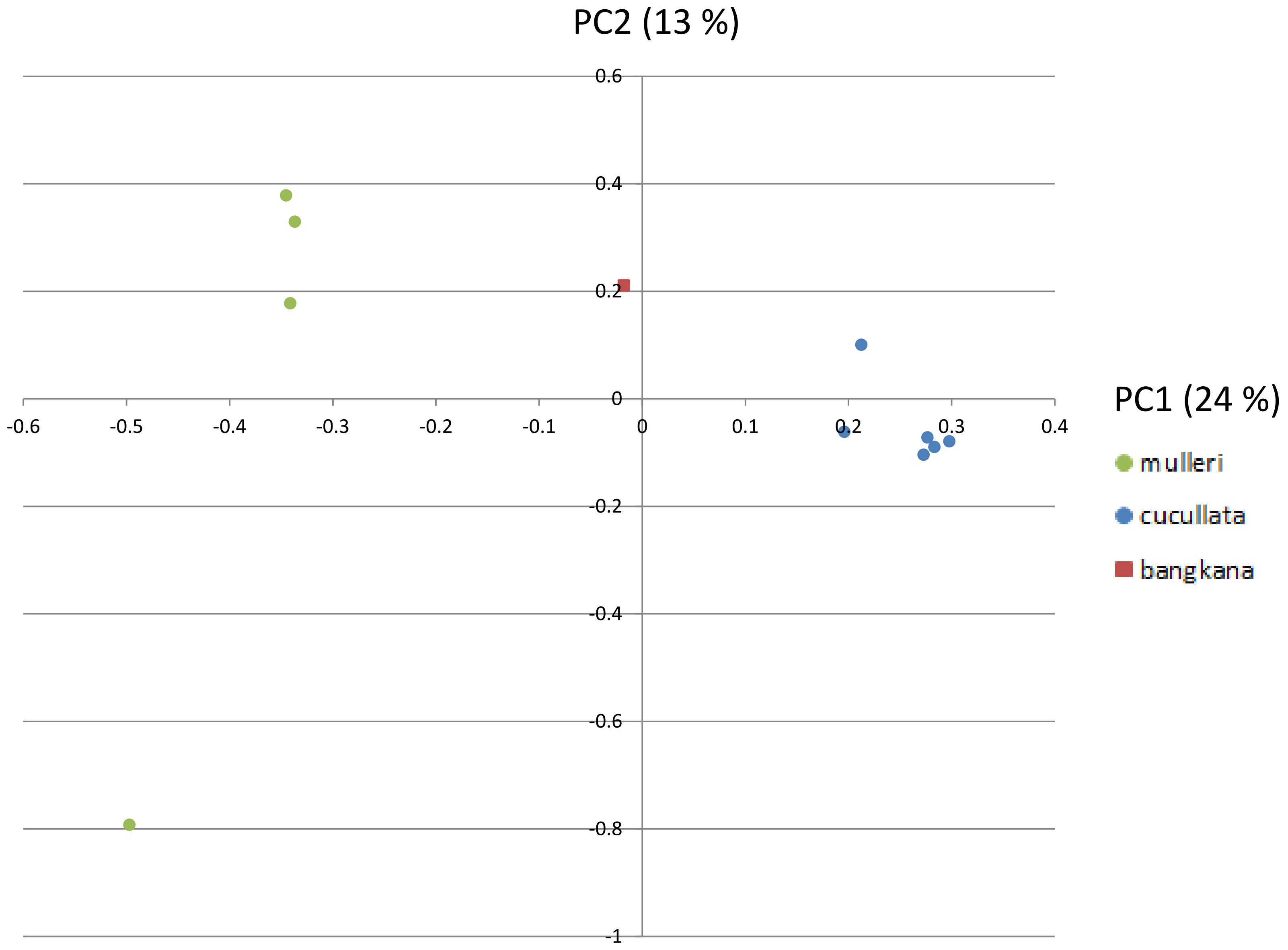

Fig. S6: Principal component analysis (PCA) of the genomic variation among the samples assigned to the taxa *mulleri*, *cucullata* and *bangkana*. Only the first to components, together representing 37% of the total variation, are shown.

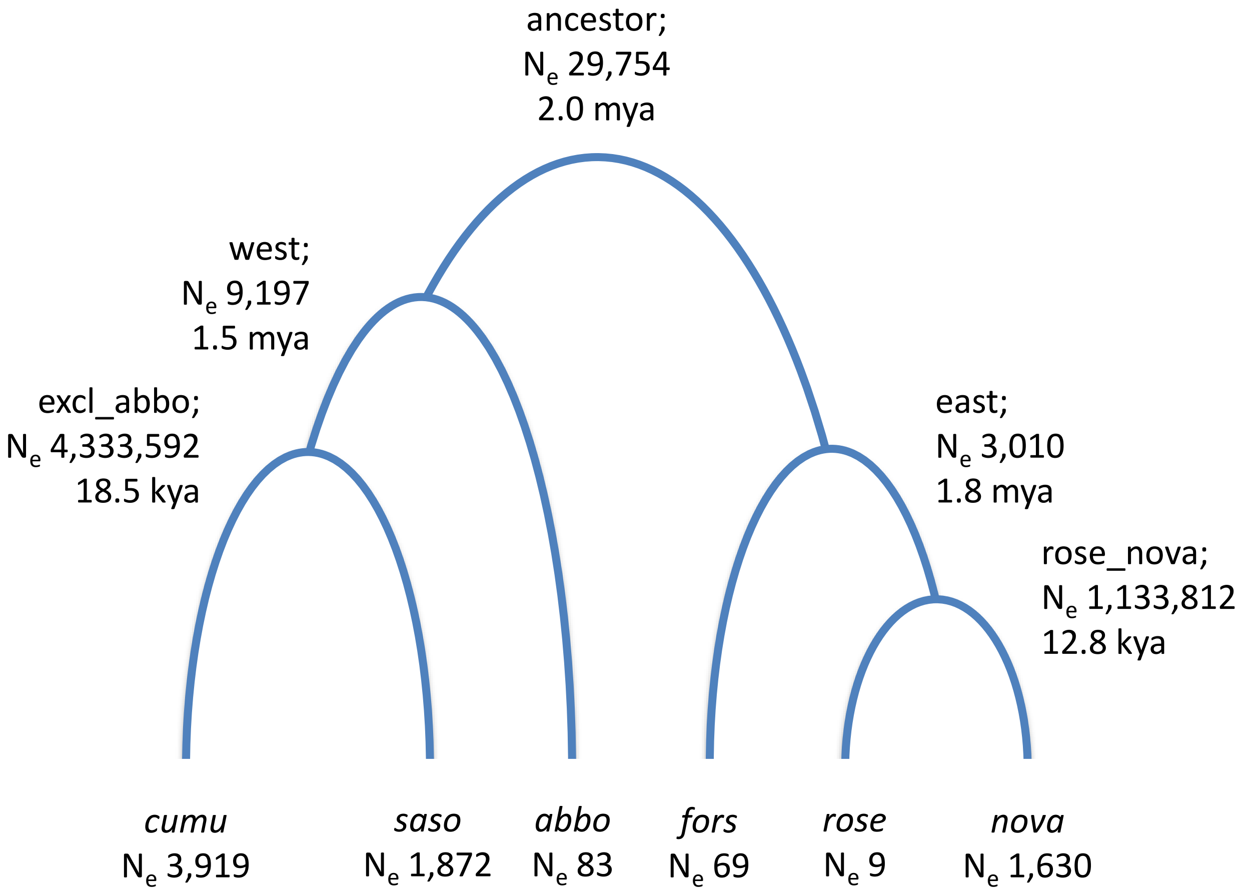

Fig. S7: Divergence times and effective population sizes (N_e) inferred by G-PhoCS using the predefined tree topology from Figure 2 (after pruning *palawanensis*, see Additional file 1). Estimated divergence times and effective population sizes are calibrated assuming an average mutation rate of $4.6*10^{-9}$ substitutions per generation and an average generation time of 4.2 year. Arrows indicate estimates of migrants per generation between lineages. See Additional file 2, Tables S3-S5 for details.

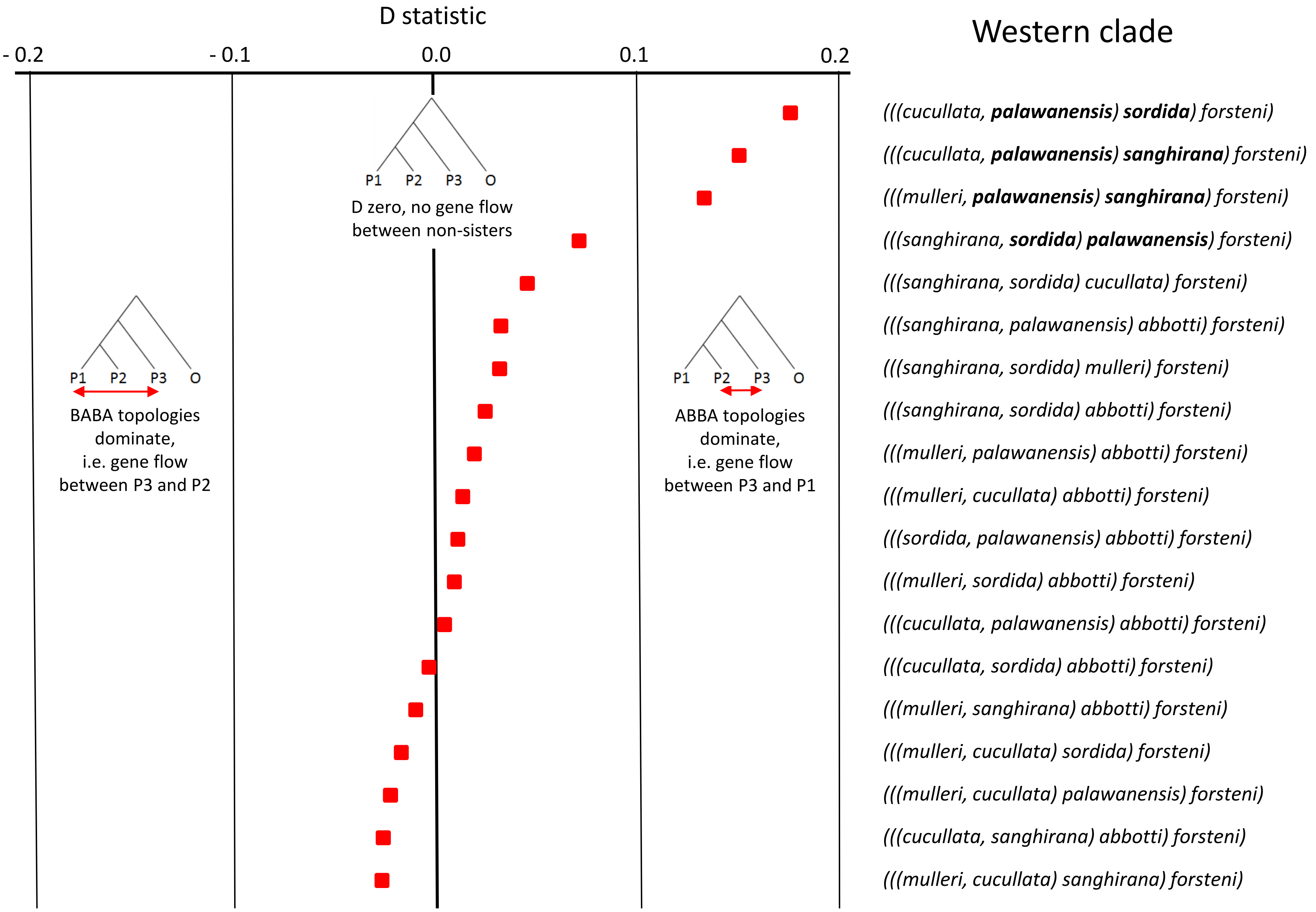

Fig. S8: D-statistic (ABBA-BABA) for the tested comparisons within the western clade. The red squares indicate D-statistic values for the complete data set. The tested phylogeny is formatted as (((P1,P2),P3),P4), where P4 is outgroup. Non-sister taxa with signatures of considerable gene flow (absolute D value > ± 0.05) are marked in bold.

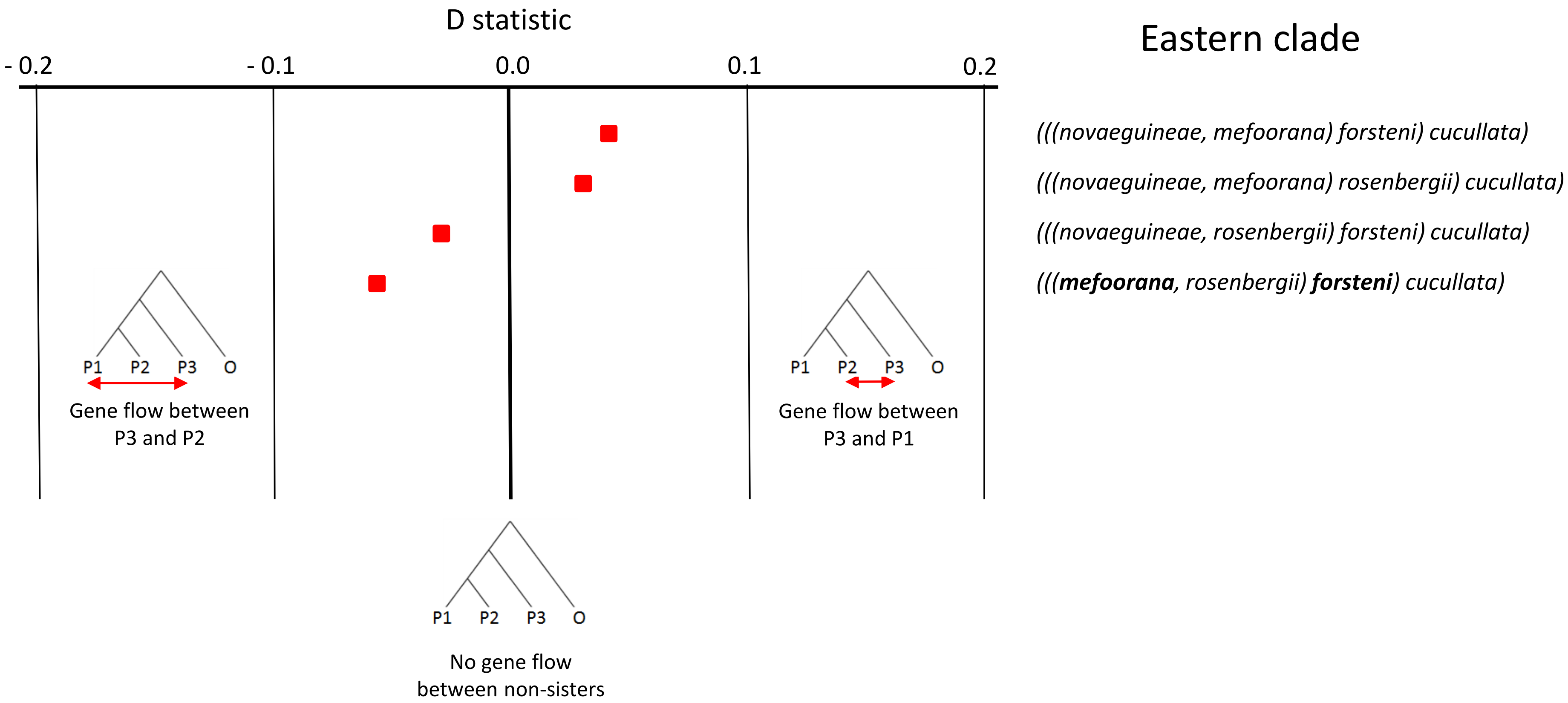

Fig. S9: D-statistic (ABBA-BABA) for the tested comparisons within the eastern clade. The red squares indicate D-statistic values for the complete data set. The tested phylogeny is formatted as (((P1,P2),P3),P4), where P4 is outgroup. Non-sister taxa with signatures of considerable gene flow (absolute D value > ± 0.05) are marked in bold.

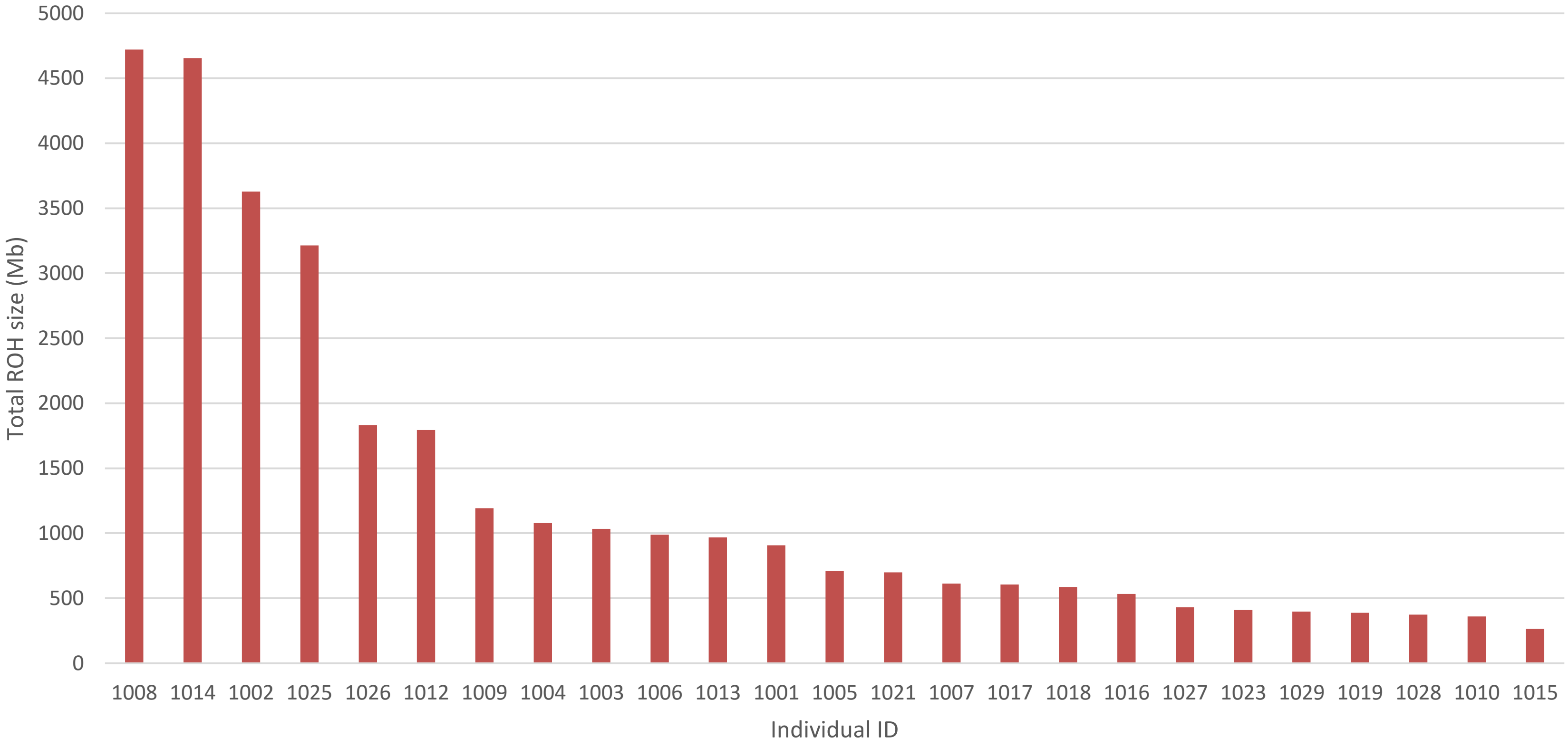

Fig. S10: Cumulative size of the genome fraction allocated in runs of homozygosity (>250 kb) per sample.

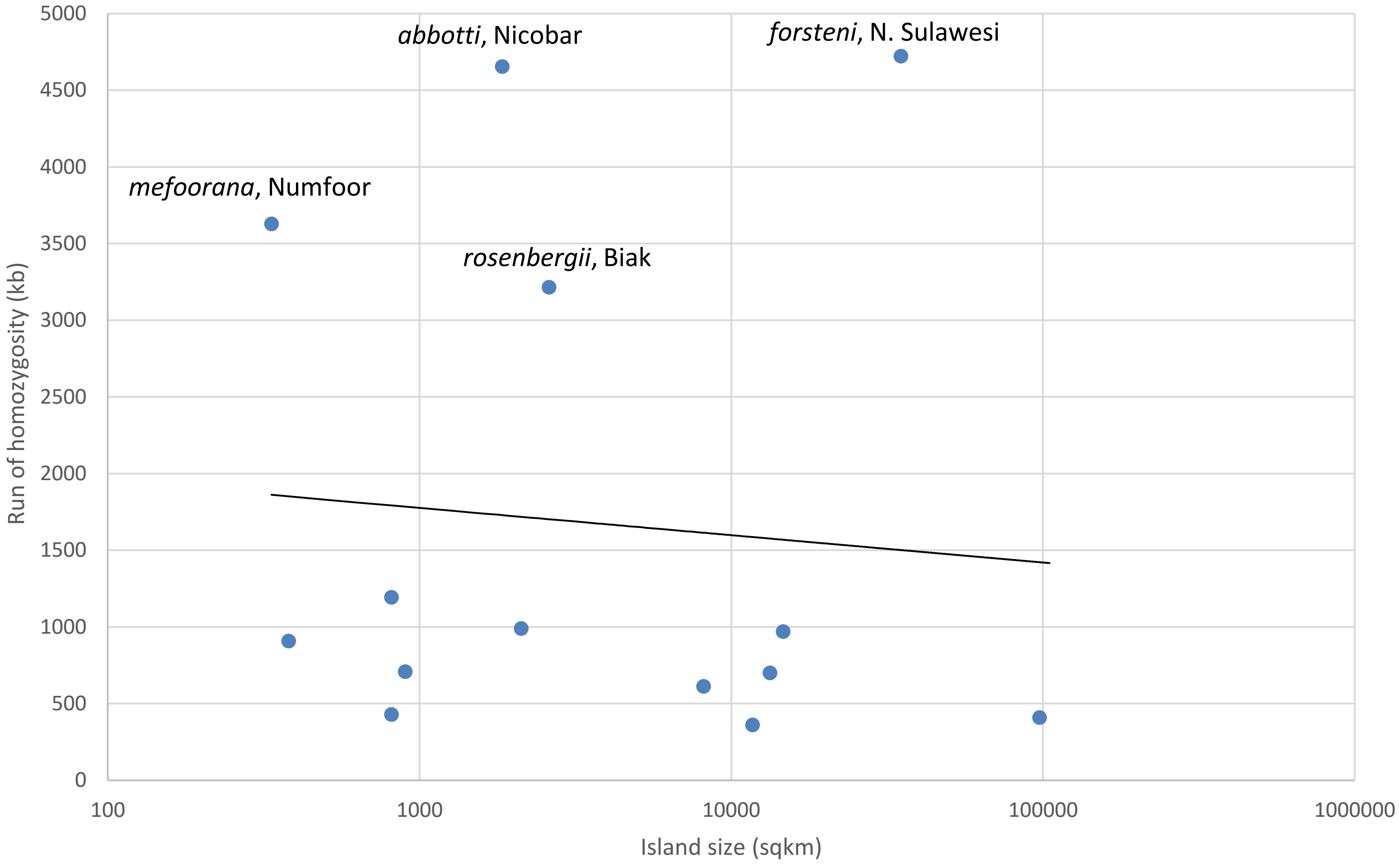

Fig. S11: Cumulative size of the genome fraction allocated in runs of homozygosity (>250 kb) plotted against the size of the island from where the sample was collected.

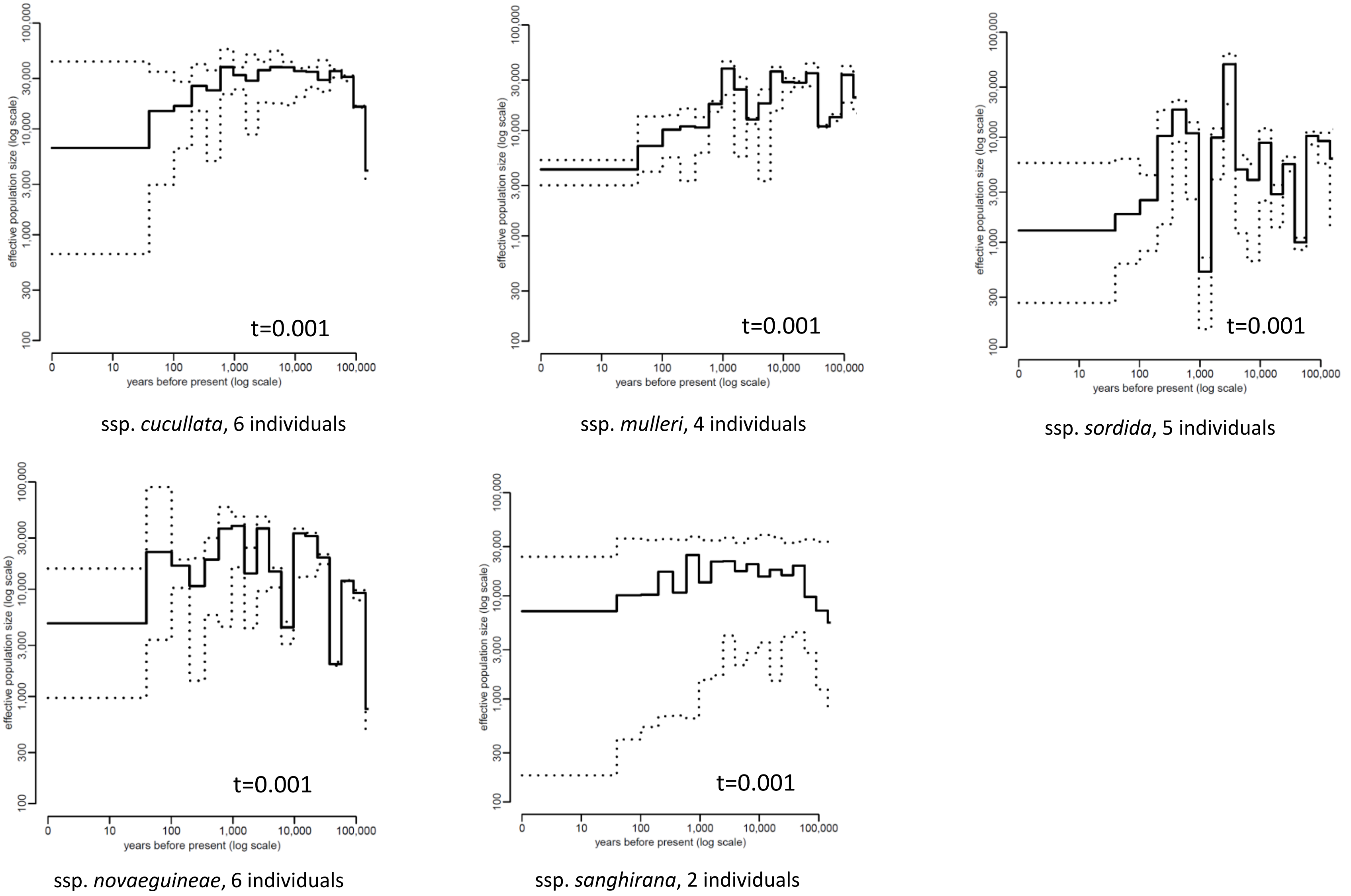

Fig. S12: Variation in effective size (Ne) in populations represented by more than one individual inferred with the approximate Bayesian computation pipeline PopSizeABC using 1,304,779 SNPs. Summary statistics of the genome-wide allele frequency spectrum (AFS) and the average zygotic linkage disequilibrium (LD) are first calculated at 21 discrete time windows, between 2,400 to 130,000 years BP, based on the empirical data set. These statistics are then compared with the corresponding statistics calculated from 400,000 simulated data sets. In the analyses was used a generation time of 4.2 year, a recombination rate of $1.0*10^{-8}$, and a genomic mutation rate per generation of $4.6*10^{-9}$.

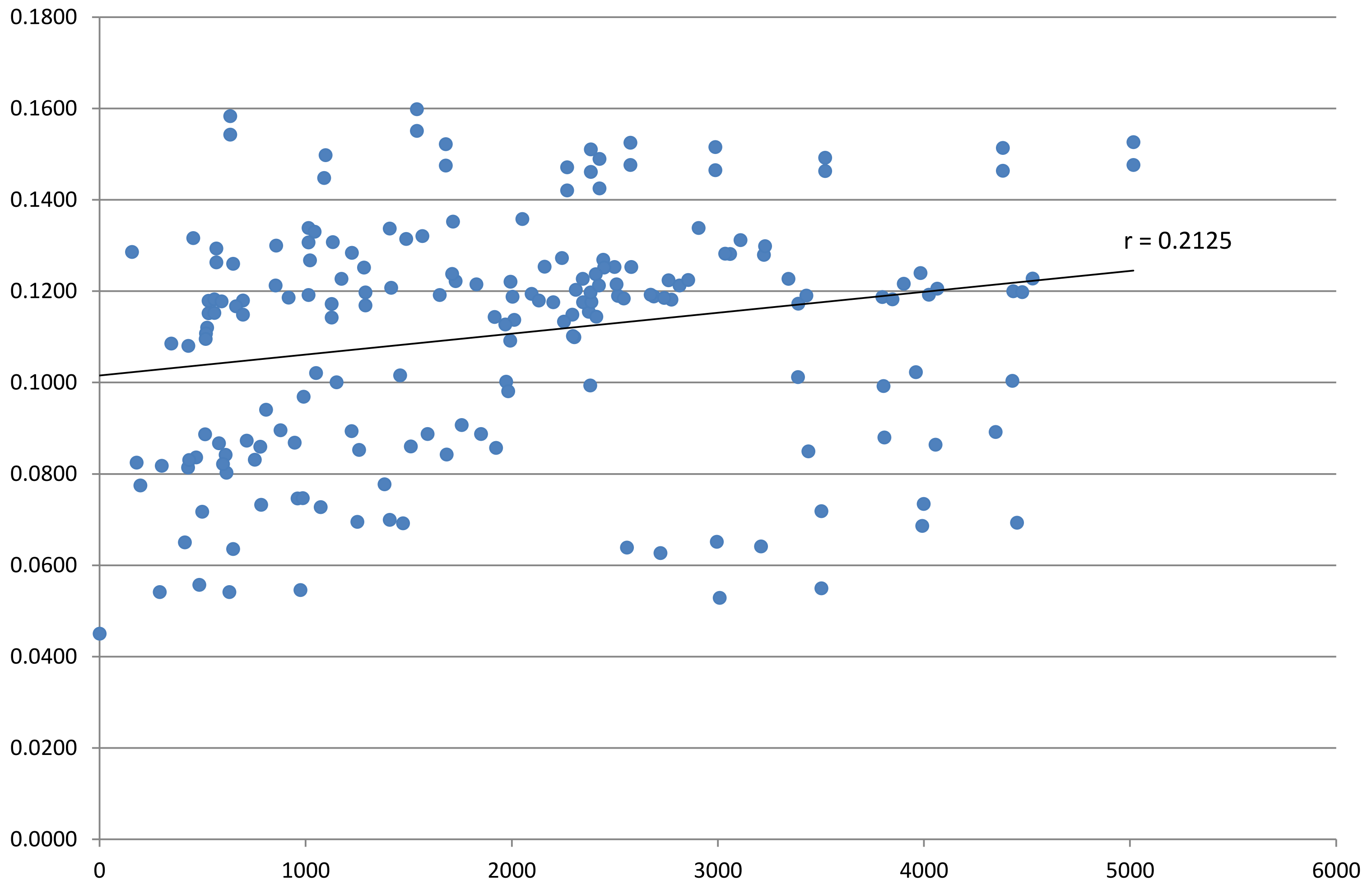

Fig. S13: Scatterplot of the genetic (p-distances) and geographic distances (km) between all pairs of samples in the western clade. Pearson's correlation coefficient r and the trend line (calculated by linear regression) that best models the data are shown in the diagram.

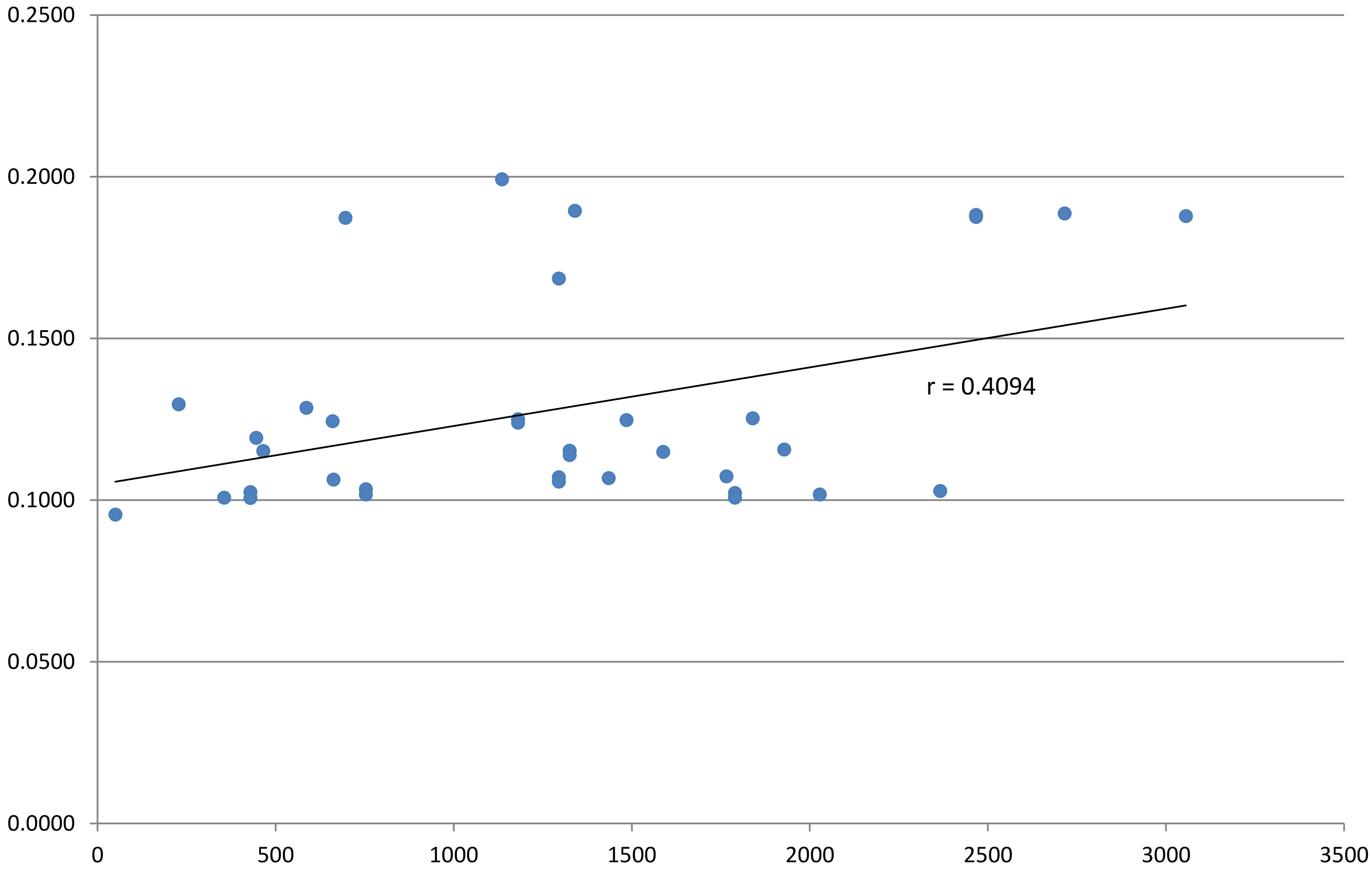

Fig. S14: Scatterplot of the genetic (p-distances) and geographic distances (km) between all pairs of samples in the eastern clade. Pearson's correlation coefficient r and the trend line (calculated by linear regression) that best models the data are shown in the diagram.

Table S1: Data sets used for reference mapping.

Abbr.	Gene name	Species	GenBank no.	Reference	Length	No. ingroup taxa	Length	MP-EST	Best model
aldob	aldolase B fructose-bisphosphate (ALDOB) gene, exons 3 through 8 and partial cds	Pitta guajana	EU737895	Hackett et al. (2008)	2'003	29	1'826	yes	TrN+I+G
bdnf	brain-derived neurotrophic factor (BDNF) gene, exon 1 and partial cds	Pitta guajana	EU738064	Hackett et al. (2008)	678	29	677	yes	TPM2uf
clct	clathrin heavy polypeptide Hc (CLTC) gene, exons 6, 7 and partial cds	Pitta guajana	EU738198	Hackett et al. (2008)	796	27	769	yes	TVM+G
cmos	oocyte maturation factor Mos (c-mos) protooncogene, partial cds	Pitta guajana	AY056952	Barker et al. (2002)	607	29	605	yes	TPM1uf+I
cryaa	crystallin alpha A (CRYAA) gene, exons 1, 2 and partial cds	Pitta guajana	EU737738	Hackett et al. (2008)	1'212	29	1'211	yes	TPM1uf+I+G
eef2	eukaryotic translation elongation factor 2 (EEF2) gene, exons 5 through 9 and partial cds	Pitta guajana	EU738675	Hackett et al. (2008)	1'811	29	1'785	yes	TPM3uf+I+G
egr1	early growth response 1 (EGR1) gene, exon 2 and partial cds	Pitta guajana	EU739005	Hackett et al. (2008)	1'197	29	1'170	yes	TVM+I+G
fgb45	fibrinogen beta chain (FGB) gene, exons 4, 5 and partial cds	Pitta guajana	EU739157	Hackett et al. (2008)	680	24	612	yes	TPM2uf
fgb68	fibrinogen beta chain (FGB) gene, exons 6 through 8 and partial cds	Pitta guajana	EU739469	Hackett et al. (2008)	1'457	28	1'428	yes	TIM2+I
g3pdh	glyceraldehyde-3-phosphate dehydrogenase (G3PDH) gene, intron 11	Pitta guajana	DQ785911	Irestedt et al. (2006)	307	28	302	yes	TIM3
hmgn2	nonhistone chromosomal protein HMG-17 (HMGN2) gene, exons 2 through 4 and partial cds	Pitta guajana	EU739562	Hackett et al. (2008)	1'884	29	1'747	yes	TrN+I+G
irbp	interphotoreceptor retinoid-binding protein (IRBP) gene, partial cds	Pitta erythrogaster	JN614758	Zuccon & Ericson (2012)	1'074	29	1'071	yes	K80+G
irf1	interferon regulatory factor 2 (IRF2) gene, intron 2	Pitta guajana	EU739704	Hackett et al. (2008)	569	27	537	yes	TVM+G
myo	myoglobin gene, intron 2, exons 2, 3 and partial cds	Pitta guajana	DQ785986	Hackett et al. (2008)	699	28	650	yes	HKY+I+G
ngf	nerve growth factor beta polypeptide (NGF) gene, partial cds and 3' UTR	Pitta guajana	EU740184	Hackett et al. (2008)	746	29	743	yes	TrN+G
ntf3	neurotrophin 3 (NTF3) gene, exon 2 and partial cds	Pitta guajana	EU740346	Hackett et al. (2008)	695	28	689	yes	TIM2+I
odc	ornithine decarboxylase (ODC) gene, introns 6, 7, exons 6, 7 and partial cds	Pitta guajana	DQ785950	Irestedt et al. (2006)	597	27	561	yes	TPM1uf+I+G
pcbd1	pterin-4 alpha-carbinolamine dehydratase (PCBD1) gene, exons 2, 3 and partial cds	Pitta guajana	EU738517	Hackett et al. (2008)	871	9	757	yes	HKY+I
rag1	recombination activating protein 1 (RAG-1) gene, partial cds	Pitta guajana	AY057021	Barker et al. (2002)	2'872	29	2'867	yes	TIM2+I
rag2	recombination activating protein 2 (RAG-2) gene, partial cds	Pitta guajana	DQ320575	Moyle et al. (2006)	1'152	29	1'151	yes	TPM2uf+G
rho	rhodopsin (RHO) gene, exons 1 through 4 and partial cds	Pitta guajana	EU737267	Hackett et al. (2008)	1'747	29	1'598	yes	TIM1+I+G
tgfb2	transforming growth factor beta 2 (TGFB2) gene, exons 5, 6 and partial cds	Pitta guajana	EU737435	Hackett et al. (2008)	597	29	540	yes	HKY+G
vmyc	v-myc myelocytomatosis viral oncogene-like protein (MYC) gene, exon 3 and partial cds	Pitta guajana	EU738351	Hackett et al. (2008)	1'244	29	1'240	yes	TPM1uf+I
mt	mitochondrial genome	Pitta nympha	KJ680302	Lee et al. (unpublished)	18'054	29	17'835	yes	TPM2uf+I+G
23 genes	concatenation of 23 nuclear genes					29	24'072		GTR+G
mt+23 genes	concatenation of mitochondrial genome + 23 nuclear genes					29	41'907		GTR+G
UCE	flanking regions sequenced after anchoring at sites for ultra-conserved elements, subsample	Pitta guajana	Dryad	McCormack et al. (2013a)	500'000	29	316'935		N/A

Table S2: Comparison of different tree topologies reconstructed in the phylogenetic analyses of different data sets and by employing different methods. Note that several topologies are incompatible with each other.

	RAxML mt genome + 23 genes	MP-EST mt genome + 23 genes	RAxML mt genome	Beast mt genome	RAxML 23 genes	MP-EST 23 genes	NJ-tree snps	Bayesian snp tree (SNAPP)
division of an Eastern and a Western group	yes (100)	yes	yes (100)	yes	yes (100)	yes	yes (100)	yes
abbotti basal to cucullata/bangkana/mulleri/ palawanensis/sordida/sanghirana	yes (100)	yes	yes (100)	yes	no	yes	yes (100)	no
sordida monophyletic	yes (100)	no	yes (100)	yes	no	no	yes (72)	yes
sordida /sanghirana monophyletic	yes (100)	yes	yes (100)	yes	no	yes	yes (100)	yes
mulleri monophyletic	yes (100)	no	yes (100)	yes	no	no	yes (100)	yes
cucullata monophyletic	yes (n.s.)	yes	no	no	no	no	yes (100)	yes
cucullata/bangkana monophyletic	yes (n.s.)	no	yes (92)	yes	no	no	yes (100)	yes
palawanensis basal to cucullata/bangkana/mulleri	yes (100)	no	yes (100)	yes	no	no	no	no
palawanensis basal to sordida/sanghirana	no	no	no	no	no	no	yes (100)	no
palawanensis basal to cucullata/bangkana/mulleri/ abbotti/sordida/sanghirana	no	no	no	no	yes (n.s.)	no	no	no
palawanensis basal to cucullata/bangkana/mulleri/ sordida/sanghirana	no	yes	no	no	no	yes	no	no
palawanensis groups with abbotti and these are basal to sordida/sanghirana	no	no	no	no	no	no	no	yes
bangkana basal to cucullata	yes (n.s.)	no	no	no	no	no	yes (100)	yes
forsteni basal to rosenbergii/mefoorana/hebetior/ novaeguineae/goodfellowi	yes (100)	yes	yes (100)	yes	yes (100)	yes	yes (100)	yes
rosenbergii basal to mefoorana/hebetior/ novaeguineae/goodfellowi	yes (100)	yes	yes (100)	yes	yes (100)	yes	yes (100)	yes
mefoorana basal to hebetior/ novaeguineae/goodfellowi	yes (n.s.)	no	yes (100)	yes	no	no	no	yes

Table S3: Calculated effective population sizes (N_e) inferred by G-PhoCS using the predefined tree topology in Figure 2 (after pruning palawanensis from the tree, see text in Additional file 1 for details) and after calibrating the estimated parameter θ assuming an average mutation rate of $4.6*10^{-9}$ substitutions per generation and an average generation length of 4.2 year.

Summary Statistic	mutation rate	theta mean	Ne (est)	95% HPD lower	95% HPD upper	95% HPD lower	95% HPD upper
theta_abbo	4.60E-09	6.39E-06	83	0.00E+00	2.00E-05	0	259
theta_cumu	4.60E-09	3.03E-04	3'919	2.00E-04	4.50E-04	2'588	5'823
theta_saso	4.60E-09	1.45E-04	1'872	9.00E-05	2.10E-04	1'165	2'717
theta_fors	4.60E-09	5.30E-06	69	0.00E+00	1.00E-05	0	129
theta_rose	4.60E-09	6.60E-07	9	0.00E+00	1.00E-05	0	129
theta_nova	4.60E-09	1.26E-04	1'630	5.00E-05	2.20E-04	647	2'847
theta_rose_nova	4.60E-09	8.76E-02	1'133'812	6.00E-02	1.18E-01	775'751	1'528'209
theta_east	4.60E-09	2.60E-04	3'368	1.20E-04	3.80E-04	1'553	4'917
theta_excl_abbo	4.60E-09	3.35E-01	4'333'592	2.45E-01	4.31E-01	3'165'114	5'571'946
theta_west	4.60E-09	7.11E-04	9'197	5.80E-04	8.20E-04	7'505	10'611
theta_ancestor	4.60E-09	2.30E-03	29'754	2.05E-03	2.53E-03	26'527	32'738

Table S4: Calculated divergence times (T) inferred by G-PhoCS using the predefined tree topology in Figure 2 (after pruning *palawanensis* from the tree, see text in Additional file 1 for details) and after calibrating the estimated parameter τ assuming an average mutation rate of $4.6*10^{-9}$ substitutions per generation and an average generation length of 4.2 year.

Summary Statistic	mutation rate	tau mean	T (est)	95% HPD lower	95% HPD upper	tau 95% lower	tau 95% upper
tau_rose_nova	4.60E-09	1.40E-05	12'773	1.00E-05	2.00E-05	9'130	18'261
tau_east	4.60E-09	2.01E-03	1'836'770	1.92E-03	2.10E-03	1'753'043	1'917'391
tau_excl_abbo	4.60E-09	2.03E-05	18'548	1.00E-05	3.00E-05	9'130	27'391
tau_west	4.60E-09	1.69E-03	1'543'226	1.63E-03	1.74E-03	1'488'261	1'588'696
tau_ancestor	4.60E-09	2.18E-03	1'987'970	2.09E-03	2.26E-03	1'908'261	2'063'478
tau_coal(estim)	4.60E-09	0.00329755	716'859				

Table S5: Estimates of mean number of migrants per generation (M_{s-t}) for the migration bands inferred by G-PhoCS using the predefined tree topology in Figure 2 (after pruning *palawanensis* from the tree, see text in Additional file 1 for details).

	m_{s-t} (estimated by G-PhoCS)	95% HPD lower	95% HPD upper	θ for the target population	$M_{s-t} = m_{s-t} \times \theta_t$ * mutation rate (= mean number of migrants per generation)	M_{s-t} 95% HPD lower	M_{s-t} 95% HPD upper
migration from (*sordida/sanghirana*) to (*cucullata/mulleri*)	1.811	1.00E-05	10.7458	3.03E-04	5.48E-04	3.03E-09	3.25E-03
migration from (*cucullata/mulleri*) to (*sordida/sanghirana*)	2.6369	1.00E-05	15.4152	1.45E-04	3.82E-04	1.45E-09	2.23E-03
migration from *rosenbergii* to *novaeguineae*	1.908	1.00E-05	10.612	1.26E-04	2.40E-04	1.26E-09	1.34E-03
migration from *novaeguineae* to *rosenbergii*	18.2012	1.00E-05	108.471	6.60E-07	1.20E-05	6.60E-12	7.16E-05

Table S6: Comparisons of estimates of divergence times for major clades within the "*Pitta sordida* species-complex" using a molecular clock approach in Beast2 and genomic mutation rates in G-PhoCS. In the G-PhoCS analysis we used two different estimates of the mutation rate in passerines; $3.0*10^{-9}$ (Zhang et al. 2014) and $4.6*10^{-9}$ (Smeds et al. 2016).

data set	mitogenomes	UCEs	UCEs
method	Beast2	G-PhoCS	G-PhoCS
mutation rate		4.60E-09	3.00E-09
generation length	N/A	4.2	4.2
	kya	kya	kya
A - Eastern vs. Western (without *palawanensis*)	3'916	1'988	3'048
B - *abbotti* vs. [*sordida, sanghirana, mulleri, cucullata, bangkana*]	1'695	1'543	2'366
C - [*sordida, sanghirana*] vs. [*mulleri, cucullata, bangkana*]	1'378	18	28
G - *forsteri* vs. [*rosenbergii*, New Guinea clade]	3'070	1'837	2'816
H - *rosenbergii* vs. New Guinea clade]	983	12	20

Table S7: Individuals and scaffolds in which longer (>250 Mb) stretches of homozygosity was observed.

ID	SNP1	SNP2	POS1	POS2	KB	NSNP	DENSITY	PHOM	PHET
1001	scaffold_2:3223402	scaffold_2:3600548	3223402	3600548	377.147	4121	0.092	0.925	0.075
1001	scaffold_0:3648704	scaffold_0:4179468	3648704	4179468	530.765	1610	0.33	0.955	0.045
1002	scaffold_51:1269632	scaffold_49:1715786	1269632	1715786	446.155	67503	0.007	0.955	0.045
1002	scaffold_10:1845862	scaffold_10:2315629	1845862	2315629	469.768	30323	0.015	0.95	0.05
1002	scaffold_6:2315643	scaffold_1:2800323	2315643	2800323	484.681	16962	0.029	0.957	0.043
1002	scaffold_4:2800427	scaffold_0:5028269	2800427	5028269	2227.843	15862	0.14	0.959	0.041
1003	scaffold_0:3653570	scaffold_0:4058619	3653570	4058619	405.05	1350	0.3	0.921	0.079
1003	scaffold_0:4259333	scaffold_0:4614436	4259333	4614436	355.104	802	0.443	0.933	0.067
1003	scaffold_0:4614673	scaffold_0:4889059	4614673	4889059	274.387	693	0.396	0.906	0.094
1004	scaffold_2:2880480	scaffold_0:3222131	2880480	3222131	341.652	6154	0.056	0.917	0.083
1004	scaffold_1:3481948	scaffold_0:3806316	3481948	3806316	324.369	1704	0.19	0.925	0.075
1004	scaffold_1:3848052	scaffold_0:4261039	3848052	4261039	412.988	1068	0.387	0.912	0.088
1005	scaffold_1:3624464	scaffold_0:4028235	3624464	4028235	403.772	1466	0.275	0.931	0.069
1005	scaffold_0:4716135	scaffold_0:5019699	4716135	5019699	303.565	821	0.37	0.948	0.052
1006	scaffold_3:3241423	scaffold_2:3592865	3241423	3592865	351.443	3725	0.094	0.92	0.08
1006	scaffold_0:4296909	scaffold_0:4614436	4296909	4614436	317.528	725	0.438	0.913	0.087
1006	scaffold_0:4614526	scaffold_0:4934647	4614526	4934647	320.122	797	0.402	0.887	0.113
1007	scaffold_0:3601652	scaffold_1:3942560	3601652	3942560	340.909	1355	0.252	0.94	0.06
1007	scaffold_0:4614673	scaffold_0:4886058	4614673	4886058	271.386	686	0.396	0.902	0.098
1008	scaffold_822:307151	scaffold_0:5028269	307151	5028269	4721.119	784718	0.006	0.975	0.025
1009	scaffold_3:3092278	scaffold_3:3402034	3092278	3402034	309.757	4555	0.068	0.934	0.066
1009	scaffold_2:3459271	scaffold_0:4341977	3459271	4341977	882.707	3249	0.272	0.949	0.051
1010	scaffold_0:3992452	scaffold_0:4352693	3992452	4352693	360.242	694	0.519	0.915	0.085
1012	scaffold_8:2766236	scaffold_2:3099639	2766236	3099639	333.404	6720	0.05	0.939	0.061
1012	scaffold_2:3564737	scaffold_0:4659218	3564737	4659218	1094.482	3255	0.336	0.941	0.059
1012	scaffold_0:4659672	scaffold_0:5025632	4659672	5025632	365.961	985	0.372	0.926	0.074
1013	scaffold_2:3421718	scaffold_0:3797081	3421718	3797081	375.364	2242	0.167	0.95	0.05
1013	scaffold_0:3797175	scaffold_0:4390737	3797175	4390737	593.563	1504	0.395	0.952	0.048
1014	scaffold_336:369020	scaffold_0:4080542	369020	4080542	3711.523	676501	0.005	0.97	0.03
1014	scaffold_0:4085274	scaffold_0:5028269	4085274	5028269	942.996	2234	0.422	0.97	0.03
1015	scaffold_0:3888460	scaffold_0:4151548	3888460	4151548	263.089	696	0.378	0.915	0.085
1016	scaffold_1:3852812	scaffold_0:4386593	3852812	4386593	533.782	1303	0.41	0.913	0.087
1017	scaffold_0:3945323	scaffold_0:4550642	3945323	4550642	605.32	1348	0.449	0.902	0.098
1018	scaffold_0:3889020	scaffold_0:4476109	3889020	4476109	587.09	1355	0.433	0.912	0.088
1019	scaffold_0:3994020	scaffold_0:4382125	3994020	4382125	388.106	754	0.515	0.899	0.101
1021	scaffold_0:3707267	scaffold_0:3958981	3707267	3958981	251.715	886	0.284	0.935	0.065
1021	scaffold_0:4069305	scaffold_0:4517019	4069305	4517019	447.715	920	0.487	0.899	0.101
1023	scaffold_0:3942942	scaffold_0:4351491	3942942	4351491	408.55	894	0.457	0.914	0.086
1025	scaffold_21:1429056	scaffold_34:1830549	1429056	1830549	401.494	50149	0.008	0.958	0.042
1025	scaffold_39:1830563	scaffold_5:2176036	1830563	2176036	345.474	24433	0.014	0.957	0.043
1025	scaffold_0:2560406	scaffold_0:2837307	2560406	2837307	276.902	8146	0.034	0.95	0.05
1025	scaffold_7:2837566	scaffold_0:5027989	2837566	5027989	2190.424	15102	0.145	0.956	0.044
1026	scaffold_0:3194861	scaffold_0:3493607	3194861	3493607	298.747	3846	0.078	0.935	0.065
1026	scaffold_1:3493807	scaffold_0:4775646	3493807	4775646	1281.84	3974	0.323	0.94	0.06
1026	scaffold_0:4777706	scaffold_0:5028269	4777706	5028269	250.564	738	0.34	0.936	0.064
1027	scaffold_7:2808558	scaffold_2:3237820	2808558	3237820	429.263	7811	0.055	0.945	0.055
1028	scaffold_0:4080796	scaffold_0:4453773	4080796	4453773	372.978	727	0.513	0.897	0.103
1029	scaffold_1:3990975	scaffold_0:4388655	3990975	4388655	397.681	772	0.515	0.921	0.079

Table S8: The *f*4-statistic was calculated in four-taxon comparisons to test different hypotheses of introgression. For each comparison, the number of biallelic SNPs used for the test and the percentage of SNPs that are variable in both pairs of sister species are given. Significance of *f*4 values was assessed with a block Jackknife procedure and with ILS-based simulations.

Hypothesis of introgression	Taxon A	Taxon B	Taxon C	Taxon D	No. SNPs	% snps variable in both population	*f*4	Probability for observed z-value after jackknifing blocks	Proportion of simulated *f*4 values larger than the observed
cumu-saso	mull_1011	cucu_1015	sord_1021	sang_1027	9'009	17.9	-0.00239	0.112	0.120
sang-sord	sang_1009	sang_1027	sord_1021	sord_1022	8'032	10.9	-0.00031	0.411	0.402
basal west	abbo_1014	pala_1013	sang_1009	sord_1021	8'442	20.7	-0.00361	0.107	0.114
nova_internal	nova1_1001	nova1_1005	nova2_1006	nova2_1007	16'952	9.4	0.00047	0.681	0.333
east-west_1	fors_1008	rose_1025	abbo_1014	sord_1021	17'627	3.3	-0.00037	0.306	0.342
east-west_2	nova_1006	nova_1007	sord_1021	sang_1027	19'728	2.0	-0.00043	0.151	0.167
east-west_3	nova_1006	fors_1008	mull_1012	sord_1024	19'397	3.2	-0.00023	0.362	0.376
east-west_4	mefo_1002	fors_1008	abbo_1014	sord_1021	18'514	3.2	0.00084	0.870	0.153
east-west_5	mefo_1002	nova_1005	sord_1021	sang_1027	19'664	2.2	-0.00057	0.089	0.156
east-west_6	nova_1005	fors_1008	abbo_1014	sord_1024	19'015	3.1	0.00112	0.941	0.096
east-west_7	nova_1005	fors_1008	abbo_1014	sang_1027	18'896	3.4	0.00094	0.882	0.160
east-west_8	nova_1005	fors_1008	abbo_1014	mull_1029	18'666	3.4	0.00122	0.946	0.079
east-west_9	nova_1005	fors_1008	cucu_1017	sord_1024	19'459	3.3	-0.00022	0.369	0.363
east-west_10	nova_1005	fors_1008	cucu_1017	sang_1027	19'354	3.4	-0.00021	0.382	0.375
east-west_11	nova_1005	fors_1008	cucu_1017	mull_1029	18'907	3.0	0.00021	0.642	0.401

Table S9: Analysis of mismatch distribution in populations within the “*Pitta sordida* species-complex” from which two or more individuals were sampled. The raggedness indices Rg (Harpending et al. 1993) and R2 (Ramos-Onsins and Rozas 2002) evaluates departure from the null-hypothesis that the population has been expanding. ns = not significant, * $p < 0.05$, ** $p < 0.01$.

			22,074 snps					41,907 nucleotides			
	n		r_g		R_2			r_g		R_2	
cucu	6		0.1511	ns	0.0973	*		0.1034	ns	0.0988	*
mull	4		0.3333	ns	0.1032	ns		0.2778	ns	0.127	ns
sang	2		2	ns	0.5	ns		2	ns	0.5	ns
sord	5		0.18	ns	0.11	*		0.18	ns	0.0933	*
nova	7		0.1043	ns	0.146	ns		0.0907	ns	0.0996	*
cucu_mull	11		0.0403	ns	0.1057	ns		0.0331	ns	0.0825	**
excl_abbo	19		0.0096	ns	0.1258	ns		0.0134	ns	0.1253	ns
sang_sord	7		0.0907	ns	0.171	ns		0.0862	ns	0.1305	ns
rose_nova	8		0.0638	ns	0.155	ns		0.0612	ns	0.1319	ns
eastern	9		0.0525	ns	0.2194	ns		0.0602	ns	0.1849	ns
western	20		0.0124	ns	0.1103	ns		0.01	ns	0.1111	ns

Table S10: Admixture signatures from D-statistic (ABBA-BABA test). Non-sister taxa with signatures of considerable gene flow (D-statistic higher than 0.05 or lower than -0.05) are marked in bold.

Western clade							
P1	P2	P3	O	nABBA	nBABA	D-statistic	Z-score
cucullata	***palawanensis***	***sordida***	*forsteni*	280'751	192'463	0.187	103.29
cucullata	***palawanensis***	***sanghirana***	*forsteni*	264'381	191'166	0.161	78.57
mulleri	***palawanensis***	***sanghirana***	*forsteni*	260'833	195'981	0.142	69.65
sanghirana	***sordida***	***palawanensis***	*forsteni*	210'343	180'080	0.078	45.00
sanghirana	*sordida*	*cucullata*	*forsteni*	185'484	168'182	0.049	32.96
sanghirana	*palawanensis*	*abbotti*	*forsteni*	173'869	161'814	0.036	16.29
sanghirana	*sordida*	*mulleri*	*forsteni*	187'133	174'232	0.036	23.82
sanghirana	*sordida*	*abbotti*	*forsteni*	152'104	144'088	0.027	15.97
mulleri	*cucullata*	*abbotti*	*forsteni*	164'469	158'855	0.017	13.84
mulleri	*palawanensis*	*abbotti*	*forsteni*	184'928	179'457	0.015	7.59
sordida	*palawanensis*	*abbotti*	*forsteni*	162'933	158'602	0.013	6.55
mulleri	*sordida*	*abbotti*	*forsteni*	185'882	184'271	0.004	2.35
cucullata	*palawanensis*	*abbotti*	*forsteni*	182'638	183'361	-0.002	-1.00
cucullata	*sordida*	*abbotti*	*forsteni*	183'035	187'141	-0.011	-5.86
mulleri	*cucullata*	*sordida*	*forsteni*	193'327	199'720	-0.016	-14.34
mulleri	*sanghirana*	*abbotti*	*forsteni*	185'717	192'386	-0.018	-8.69
mulleri	*cucullata*	*palawanensis*	*forsteni*	186'251	195'190	-0.023	-18.84
mulleri	*cucullata*	*sanghirana*	*forsteni*	188'424	198'608	-0.026	-21.27
cucullata	*sanghirana*	*abbotti*	*forsteni*	183'592	196'113	-0.033	-16.037
Eastern clade							
P1	P2	P3	O	nABBA	nBABA	D-statistic	Z-score
novaeguineae	*mefoorana*	*rosenbergii*	*cucullata*	89'929	86'577	0.019	6.120
novaeguineae	*novaeguineae*	*forsteni*	*cucullata*	40'859	40'150	0.009	2.181
novaeguineae	*novaeguineae*	*rosenbergii*	*cucullata*	79'456	78'642	0.005	1.629
novaeguineae	*novaeguineae*	*mefoorana*	*cucullata*	115'751	118'674	-0.012	-3.921
novaeguineae	*rosenbergii*	*forsteni*	*cucullata*	47'326	49'553	-0.023	-6.126
mefoorana	*novaeguineae*	*rosenbergii*	*cucullata*	83'406	88'119	-0.027	-8.844
mefoorana	*novaeguineae*	*forsteni*	*cucullata*	41'822	45'035	-0.037	-8.727
mefoorana	*rosenbergii*	***forsteni***	*cucullata*	46'409	52'286	-0.060	-12.41

Table S11: Alternative taxonomic classifications of the taxa included in the "*Pitta sordida* species-complex".

	Mayr (1979)	Lambert & Woodcock (1996)	Erritzoe & Erritzoe (1998)	Erritzoe (2003) - HBW	HBW Alive
abbotti	Hooded Pitta (*Pitta sordida abbotti*)	Hooded Pitta (*Pitta sordida abbotti*)	Hooded Pitta (*Pitta sordida abbotti*)	Hooded Pitta (*Pitta sordida abbotti*)	Western Hooded Pitta (*Pitta sordida abbotti*)
cucullata	Hooded Pitta (*Pitta sordida cucullata*)	Hooded Pitta (*Pitta sordida cucullata*)	Hooded Pitta (*Pitta sordida cucullata*)	Hooded Pitta (*Pitta sordida cucullata*)	Western Hooded Pitta (Pitta sordida cucullata)
bangkana	Hooded Pitta (*Pitta sordida bangkana*)	Hooded Pitta (*Pitta sordida bangkana*)	Hooded Pitta (*Pitta sordida bangkana*)	Hooded Pitta (*Pitta sordida bangkana*)	Western Hooded Pitta (*Pitta sordida bangkana*)
mulleri	Hooded Pitta (*Pitta sordida mulleri*)	Hooded Pitta (*Pitta sordida mulleri*)	Hooded Pitta (*Pitta sordida mulleri*)	Hooded Pitta (*Pitta sordida mulleri*)	Western Hooded Pitta (*Pitta sordida mulleri*)
palawanensis	Hooded Pitta (*Pitta sordida palawanensis*)	Hooded Pitta (*Pitta sordida palawanensis*)	Hooded Pitta (*Pitta sordida palawanensis*)	Hooded Pitta (*Pitta sordida palawanensis*)	Western Hooded Pitta (*Pitta sordida palawanensis*)
sordida	Hooded Pitta (*Pitta sordida sordida*)	Hooded Pitta (*Pitta sordida sordida*)	Hooded Pitta (*Pitta sordida sordida*)	Hooded Pitta (*Pitta sordida sordida*)	Western Hooded Pitta (*Pitta sordida sordida*)
sanghirana	Hooded Pitta (*Pitta sordida sanghirana*)	Hooded Pitta (*Pitta sordida sanghirana*)	Hooded Pitta (*Pitta sordida sanghirana*)	Hooded Pitta (*Pitta sordida sanghirana*)	Western Hooded Pitta (*Pitta sordida sanghirana*)
Forsteni	Hooded Pitta (*Pitta sordida forsteni*)	Hooded Pitta (*Pitta sordida forsteni*)	Hooded Pitta (*Pitta sordida forsteni*)	Hooded Pitta (*Pitta sordida forsteni*)	Western Hooded Pitta (*Pitta sordida forsteni*)
rosenbergii	Hooded Pitta (*Pitta sordida rosenbergii*)	Hooded Pitta (*Pitta sordida rosenbergii*)	Hooded Pitta (*Pitta sordida rosenbergii*)	Hooded Pitta (*Pitta sordida rosenbergii*)	Biak Hooded Pitta (*Pitta rosenbergii*)
mefoorana	Hooded Pitta (*Pitta sordida mefoorana*)	Hooded Pitta (*Pitta sordida mefoorana*)	Hooded Pitta (*Pitta sordida mefoorana*)	Hooded Pitta (*Pitta sordida mefoorana*)	Eastern Hooded Pitta (*Pitta novaeguineae mefoorana*)
hebetior	Hooded Pitta (*Pitta sordida hebetior*)	Hooded Pitta (*Pitta sordida novaeguineae*)	Hooded Pitta (*Pitta sordida hebetior*)	Hooded Pitta (*Pitta sordida hebetior*)	Eastern Hooded Pitta (*Pitta novaeguineae novaeguineae*)
novaeguineae	Hooded Pitta (*Pitta sordida novaeguineae*)	Hooded Pitta (*Pitta sordida novaeguineae*)	Hooded Pitta (*Pitta sordida novaeguineae*)	Hooded Pitta (*Pitta sordida novaeguineae*)	Eastern Hooded Pitta (*Pitta novaeguineae novaeguineae*)
goodfellowi	Hooded Pitta (*Pitta sordida goodfellowi*)	Hooded Pitta (*Pitta sordida goodfellowi*)	Hooded Pitta (*Pitta sordida goodfellowi*)	Hooded Pitta (*Pitta sordida goodfellowi*)	Eastern Hooded Pitta (*Pitta novaeguineae goodfellowi*)

Table S12: Specimen data for the samples used in the study. Acronyms: AMNH, American Museum of Natural History; ZMA, Zoological Museum Amsterdam (Naturalis, Leiden); LKCNHM, Lee Kong Chian Natural History Museum Singapore.

Sample no.	Taxon	Specimen no.	Locality on label	Collector	Date collected	Age	Sex
1001	*hebetior*	AMNH 554058	Dampier I., German New Guinea	AS Meek	1914-02-09	adult	male
1002	*mefoorana*	AMNH Stein 871	Mafor (=Numfor)	Stein	1931-04-11	adult	female
1003	*novaeguineae*	AMNH 553990	Milne Bay, SE New Guinea	Meek	1899-04-01	adult	male
1004	*novaeguineae*	AMNH 553995	Kumusi R, NE New Guinea	Meek	1909-06-23	adult	male
1005	*novaeguineae*	AMNH 838604	Siassi Arch, Crown I	Diamond	1972-08-06	adult	female
1006	*novaeguineae*	AMNH 554036	Misol	Kuhn	1900-02-02	adult	male
1007	*goodfellowi*	AMNH 554042	Trangan I, Aru Is		1900-09-21	adult	male
1008	*forsteni*	AMNH 299358	Kumasot? Minahassa, 250 m, N. Celebes	Heinrich	1931-02-25	adult	male
1009	*sanghirana*	AMNH 554148	Gros Sangir	CWC	1893-07-18	adult	unknown
1010	*bangkana*	AMNH 554122	Simpang, Bangka	Hagen	May 1905	subadult	female
1011	*mulleri*	AMNH 447701	Riam, Kohawerniger?, SW Borneo	Menden	1935-12-08	adult	female
1012	*mulleri*	AMNH 554109	Sibutu I	Everett	July 1893	adult	male
1013	*palawanensis*	AMNH 468177	Aborlan, Palawan	Celestino	1955-05-08	adult	female
1014	*abbotti*	AMNH 554140	Great Nicobar	BB Osmaston	1905-04-10	adult	female
1015	*cucullata*	AMNH 777557	Hetora, Nepal	Koelz	1947-05-20	adult	female
1016	*cucullata*	AMNH 110600	Baldamgiri, Assam, India	Crozier	1906-10-10	adult	female
1017	*cucullata*	AMNH 554131	Gunung Tahan, Malaysia		February 1902	adult	female
1018	*mulleri*	AMNH 554124	Djampang, Java, 1500 ft		March 1898	adult	
1019	*mulleri*	AMNH 554123	Toenboenga?, Deli, Sumatra	van Heyst	1917-12-18	adult	male
1020	*sordida*	AMNH 296057	Luzon, San Pedro Tunasan?, Laguna	Zimmer	1916-10-02	adult	male
1021	*sordida*	AMNH 790583	Negros, Bagacay, Dumaguete City	Rabor	1958-01-27	adult	female
1022	*sordida*	AMNH 790113	Mindoro, Mt. Halcon	Ramos	1965-03-16	adult	female
1023	*sordida*	AMNH 554102	Davao, Mindanao	Goodfellow	April 1903a	adult	female
1024	*sordida*	AMNH 554106	Jolo, Sulu	Platen	1887-05-24	adult	male
1025	*rosenbergii*	ZMA.AVES.30052	Irian Jaya, New Guinea	Frank jr, G.A.			
1026	*cucullata*	LKCNHM WLK652	Singapore, Bukit Batok Nature Park	Frank Rheindt	2008-02-28		
1027	*sanghirana*	AMNH554145	Gros Sangir	Platen	1887-01-29	adult	male
1028	*mulleri*	AMNH805920	Majong? River, Sarawak, Borneo	Kuser-Beebe	9 July 1910 (or 7 Sep)	juvenile	female
1029	*mulleri*	AMNH554114	Labuan, N Borneo	Whitehead	1886-06-15	adult	female

Table S13: Mapping coverage estimated with genomecov in BEDTools v.2.26.0.

		Mt genome 18,054 bp		23 nuclear 25,495 bp		UCE:s 316,935 bp		Genome 1,100,385,636 bp	
		depth	breadth	depth	breadth	depth	breadth	depth	breadth
1001	hebetior AMNH554058	413	0.95	112	0.92	3.6	0.94	5.2	0.91
1002	mefoorana AMNH Stein 871	251	0.94	215	0.94	3.6	0.94	5.6	0.91
1003	novaeguineae AMNH 553990	429	0.94	113	0.93	2.9	0.90	4.4	0.89
1004	novaeguineae AMNH 553995	301	0.95	116	0.90	2.7	0.89	4.1	0.88
1005	novaeguineae AMNH 838604	1561	0.96	217	0.98	5.5	0.99	6.9	0.93
1006	novaeguineae AMNH 554036	306	0.94	140	0.93	3.3	0.92	4.9	0.90
1007	goodfellowi AMNH 554042	1775	0.97	100	0.91	2.7	0.90	3.9	0.88
1008	forsteni AMNH 299358	547	0.97	186	0.85	1.5	0.70	3.6	0.78
1009	sanghirana AMNH 554148	272	0.99	121	0.87	1.8	0.74	3.5	0.82
1010	bangkana AMNH 554122	246	0.99	140	0.95	5.7	0.98	7.0	0.95
1011	mulleri AMNH 447701	630	0.99	179	0.89	2.4	0.83	4.6	0.86
1012	mulleri AMNH 554109	898	0.99	112	0.72	1.0	0.56	2.6	0.68
1013	palawanensis AMNH 468177	589	0.99	254	0.94	4.1	0.94	6.9	0.93
1014	abbotti AMNH 554140	767	0.99	183	0.88	2.2	0.78	4.4	0.83
1015	cucullata AMNH 777557	529	0.99	295	0.96	4.3	0.93	7.8	0.92
1016	cucullata AMNH 110600	524	0.99	160	0.93	3.3	0.90	5.5	0.91
1017	cucullata AMNH 554131	774	0.99	216	0.92	3.3	0.91	6.2	0.91
1018	cucullata AMNH 554124	701	0.99	314	0.93	3.7	0.93	6.7	0.92
1019	cucullata AMNH 554123	1083	0.99	220	0.91	2.7	0.82	5.7	0.86
1020	sordida AMNH 296057	595	0.99	215	0.90	2.3	0.77	5.1	0.83
1021	sordida AMNH 790583	1740	0.99	195	0.93	3.9	0.94	7.0	0.92
1022	sordida AMNH 790113	438	0.99	179	0.96	5.5	0.98	6.9	0.94
1023	sordida AMNH 554102	355	0.99	198	0.89	2.1	0.73	4.7	0.80
1024	sordida AMNH 554106	370	0.99	170	0.94	3.1	0.88	5.4	0.88
1025	rosenbergii ZMA.AVES.30052	709	0.95	243	0.94	4.0	0.92	10.4	0.90
1026	cucullata LKCNHM WLK652	901	0.99	250	0.97	9.4	1.00	9.3	0.96
1027	sanghirana AMNH554145	1468	1.00	166	0.95	6.2	0.99	7.8	0.95
1028	mulleri AMNH805920	7296	0.99	160	0.85	1.7	0.76	3.3	0.76
1029	mulleri AMNH554114	2469	0.99	287	0.92	3.3	0.91	6.8	0.91
